# Triarylborane Dyes as a Novel Non-Covalent and Non-Inhibitive Fluorimetric Markers for DPP III Enzyme

**DOI:** 10.3390/molecules26164816

**Published:** 2021-08-09

**Authors:** Željka Ban, Zrinka Karačić, Sanja Tomić, Hashem Amini, Todd B. Marder, Ivo Piantanida

**Affiliations:** 1 Division of Organic Chemistry & Biochemistry, Ruđer Bošković Institute, P.O. Box 180, HR-10002 Zagreb, Croatia; Zeljka.Ban@irb.hr (Ž.B.); zkaracic@irb.hr (Z.K.); 2 Institut für Anorganische Chemie and Institute for Sustainable Chemistry & Catalysis with Boron, Julius-Maximilians-Universität Würzburg, 97074 Würzburg, Germany; hashem.amini@tamu.edu (H.A.); todd.marder@uni-wuerzburg.de (T.B.M.)

**Keywords:** triarylborane, pyrene, click CuAAC synthesis, DPP III enzyme, BSA, fluorescence

## Abstract

Novel dyes were prepared by simple “click CuAAC” attachment of a triarylborane–alkyne to the azide side chain of an amino acid yielding triarylborane dye **1** which was conjugated with pyrene (dye **2**) forming a triarylborane–pyrene FRET pair. In contrast to previous cationic triarylboranes, the novel neutral dyes interact only with proteins, while their affinity to DNA/RNA is completely abolished. Both the reference triarylborane amino acid and triarylborane–pyrene conjugate bind to BSA and the hDPP III enzyme with high affinities, exhibiting a strong (up to 100-fold) fluorescence increase, whereby the triarylborane–pyrene conjugate additionally retained FRET upon binding to the protein. Furthermore, the triarylborane dyes, upon binding to the hDPP III enzyme, did not impair its enzymatic activity under a wide range of experimental conditions, thus being the first non-covalent fluorimetric markers for hDPP III, also applicable during enzymatic reactions with hDPP III substrates.

## 1. Introduction

Within the last few years, triarylborane derivatives which are stable in water have been developed, and have emerged as novel fluorophores for biochemical and biological applications [1,2,3,4,5,6]. A more detailed search of their bio-relevant target molecule revealed a very rare propensity, namely that bis-triarylborane cations bind exceptionally strongly (in nM affinity) to very different targets: DNA, RNA, and protein BSA (bovine serum albumin, the most abundant protein in blood plasma responsible for the transport of many small molecules [7]), some of them strongly distinguishing by a different fluorescence response between DNA/RNAs and protein BSA [6]. However, in recent studies, we found that omitting the positive charge on trimethylammonium (NMe_3_) groups at the *para*-position aryls attached to boron completely abolishes triarylborane interactions with DNA/RNA, thus, the neutral triarylborane core binds exclusively to proteins with high (50–10 nM) affinity [8,9]. Such neutral conjugated systems consisting of an NMe_2_ donor and the 3-coordinate boron acceptor [1] result in charge transfer transitions and, consequently, strong bathochromic shifts of the UV/Vis and fluorescence emission spectra with respect to cationic analogues (see [8]), which is more convenient for some fluorescence bioimaging applications. To exploit such a strong protein affinity for inhibition of biorelevant processes, we looked for an enzyme characterized by broad substrate adaptability and a hydrophobic active site and of adequate size and shape for a voluminous triarylborane core (e.g., resembling the previously proposed binding site in BSA protein [6]).

One such enzyme is dipeptidyl peptidase III (DPP III), a cytosolic enzyme belonging to the metallopeptidase family M49 [10] that cleaves dipeptides from the N-termini of its oligopeptide substrates. DPP III is broadly distributed in mammalian tissues and thought to contribute to the final steps of normal intracellular protein catabolism, although its exact role(s) are still unclear. DPP III shows pronounced affinity for some bioactive peptides (angiotensins II, III, IV, and opioid peptides), and also flavonoids [11,12], as well as for many different peptidomimetics, many of which contain large aromatic moieties (naphthalene, phenanthridine, cyanine, pyrene, etc.) [13,14]. The wide substrate specificity of human DPP III could be attributed to the high flexibility of its 3D structure and plasticity of its ligand-binding site. Increased amounts and activity of DPP III compared to that in normal tissue indicate involvement of DPP III in the development of some cancers in humans [15,16]. Relatively recently, the potential role of DPP III in the regulation of the Nrf2/KEAP1 signaling pathway was found [17] and the mechanism of binding of human DPP III to Keap1 was elucidated [18]. Thus, the development of effective fluorescent inhibitors of human DEPP III would be of significant importance in revealing its physiological role.

In the design of a novel triarylborane probe, which could be applied for DPP III binding, we wanted to have broad structural adaptability of the eventual probe and, therefore, we synthesized a triarylborane–amino acid conjugate with free N- and C-termini. Such a versatile form of triarylborane fluorophore would allow its easy incorporation into any position of various peptidoids of interest for DPP III substrate inhibitor design. Furthermore, based on previous good results with pyrene-based DPP III inhibitors [13,14], we also prepared a triarylborane–amino acid–pyrene conjugate, such construct intrinsically containing possible Förster resonance energy transfer (FRET) between pyrene (*λ*_exc_ 342 nm/*λ*_em_ 400–450 nm) and triarylborane (*λ*_exc_ 400 nm/*λ*_em_ 540 nm) fluorophores. Pyrene’s intrinsic fluorescence is highly sensitive to changes in the polarity of its micro-environment and thus can exhibit various fluorescent characteristics: excimer formation (interaction between two pyrenes) and FRET (interactions with another fluorophore). Förster resonance energy transfer (FRET) is an important photophysical technique widely employed for monitoring molecular distances and interactions at the nano-scale level. In the process of FRET, initially, a donor fluorophore absorbs energy due to the excitation by incident light and transfers its excitation energy to a nearby acceptor chromophore via a short-range (≤10 nm) dipole–dipole interaction [19]. As FRET depends strongly on the distance and relative orientation between the donor and acceptor, it enables investigations of structure and dynamics in living cells [20,21] and can be an important strategy for the development of new fluorescent probes and sensors.

**Scheme 1:**
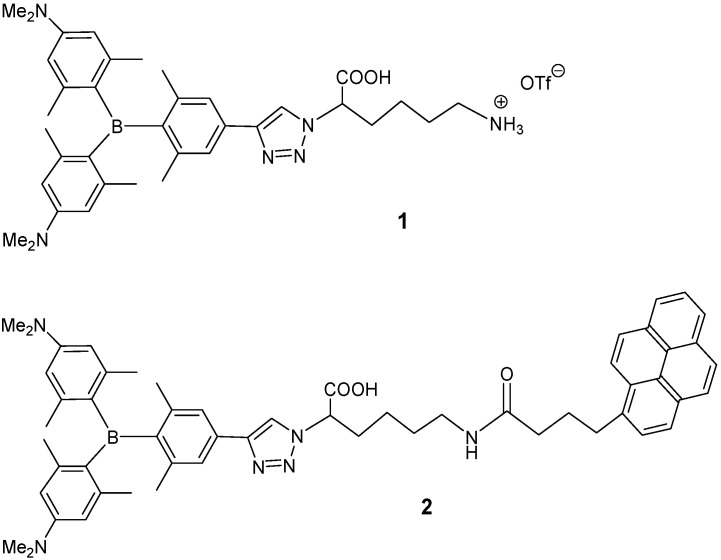
Two novel peptide-based triarylborane derivatives **1** and **2**.

For the preparation of the novel hybrid compounds **1** and **2** (Scheme 1), we have chosen a “click CuAAC” (Copper (I) catalyzed azide–alkyne cycloaddition reaction) [22,23] chemistry approach for several reasons. This coupling method allows, in the future, additional orthogonal approaches to novel peptides by introducing the triarylborane probe at the most convenient time (before or after peptide synthesis). In addition, 1,2,3-triazoles, as strong H-bond acceptors, readily interact with different biological targets through hydrogen bonding, as well as dipole and π–π stacking interactions. In addition, triazoles themselves show diverse anticancer [24], antibacterial [25], and antiviral biological activities [26].

## 2. Results and Discussion

### 2.1. Synthesis of 1,4-Disubstituted-1,2,3-triazole Triarylborane-Amino Acid Conjugates

**Scheme 2:**
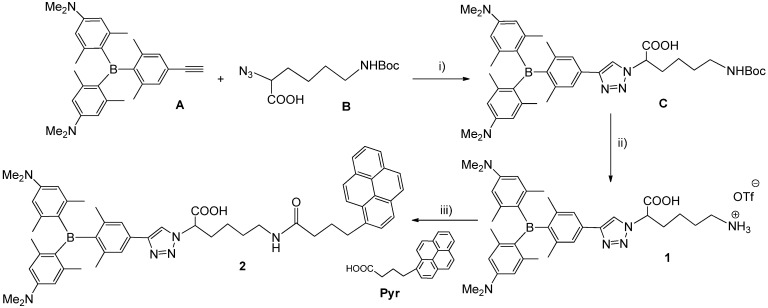
Synthesis of conjugates **1** and **2**. Reagents and conditions: (i) CuI, DIPEA, DMF, room temp., 16 h, 85%; (ii) TFA/CH_2_Cl_2_, room temp., 3 days, 100%; (iii) HBTU, HOBt, Et_3_N, DMF, room temp., 16 h, 40%.

The synthetic route for compounds **1** and **2** is depicted in Scheme 2. First, triarylborane alkyne **A** and the azido derivative of lysine amino acid **B** were prepared according to the literature [8,27]. Cu(I)-catalyzed 1,3-dipolar cycloaddition of **A** with **B** in anhydrous DMF and DIPEA at room temperature afforded conjugate **C** in good yield. Deprotection of the Boc protecting group of compound **C** was performed by a classical method in TFA/CH_2_Cl_2_, and triazole-linked triarylborane amino acid conjugate **1** was isolated in quantitative yield as a yellow powder. A standard coupling reaction between pyrene butyric acid (Pyr) and the conjugate **1** using HBTU/HOBt as the coupling reagents and triethylamine in DMF [28] gave the 1,2,3-triazole-linked triarylborane–aminoacid–pyrene conjugate **2** as a yellow powder.

### 2.2. Photophysical Properties

#### 2.2.1. Absorption and Emission Spectra

Cationic compound **1** was soluble in water, allowing preparation of stock solution (*c* = 1 × 10^−3^ mol dm^−3^), while neutral compound **2** was dissolved in DMSO to obtain a stock solution (*c* = 1 × 10^−3^ mol dm^−3^). Absorbances of the dyes were proportional to their concentrations up to 2 × 10^−5^ mol dm^−3^, and increasing the temperature did not alter the UV/Vis or emission spectra significantly (Supplementary Information, Figures S1 and S2), thus indicating that the molecules do not aggregate under experimental conditions, nor are intramolecular interactions between two chromophores present in **2**. The corresponding absorption maxima and molar extinction coefficients are given in Table 1.

**Figure 1:**
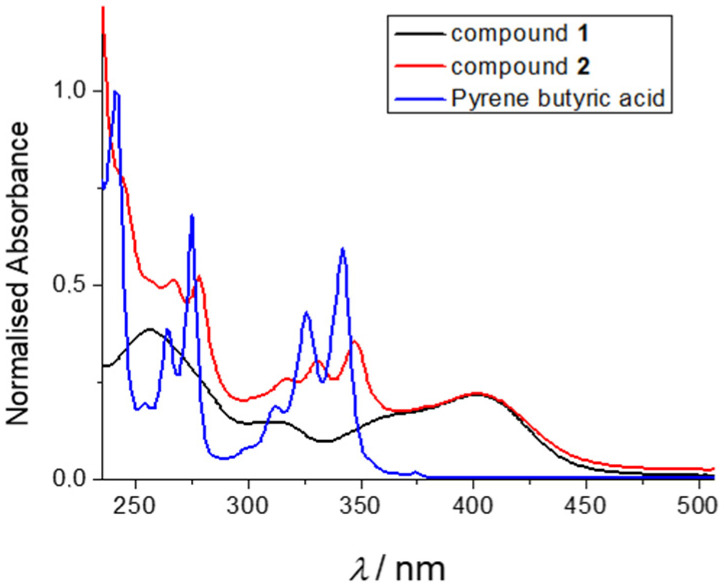

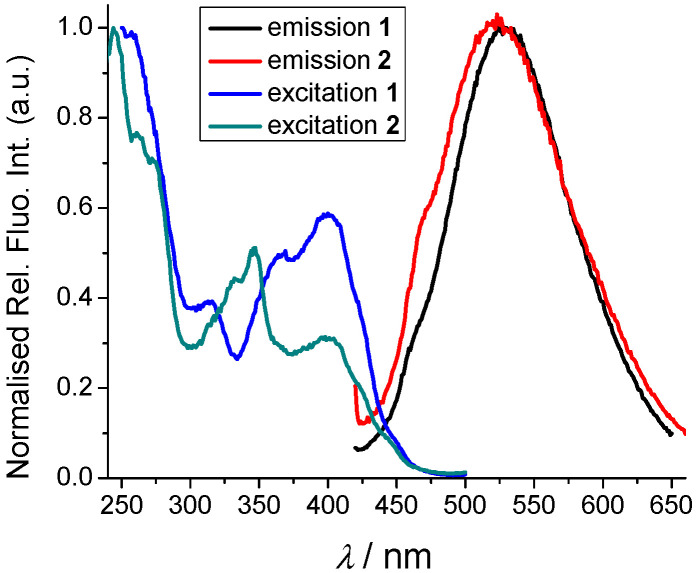
Normalised UV/Vis (Top: *c* = 2 × 10^−5^ mol dm^−3^) and fluorescence excitation and emission spectra (bottom: *c* = 3 × 10^−6^ mol dm^−3^) of **1** (λ_exc_ = 400 nm) and **2** (λ_exc_ = 403 nm) at pH 7, sodium cacodylate buffer, I = 0.05 mol dm^−3^. Note the good agreement of UV/Vis and excitation spectra.

**Figure 2:**
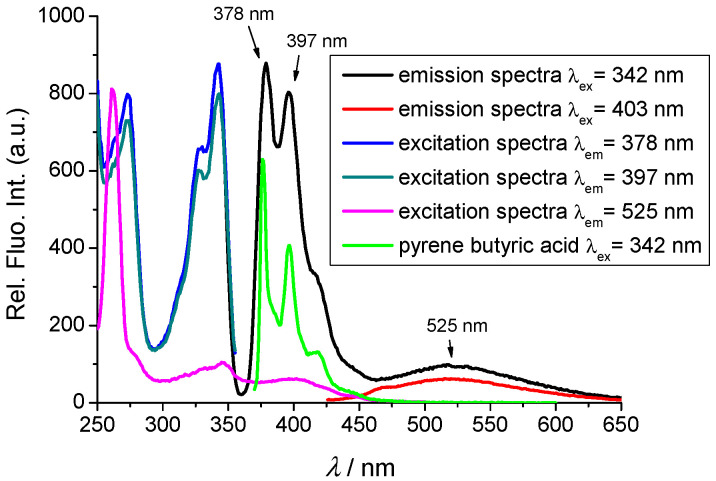
Comparison of emission and excitation spectra of **2** (λ_exc_ = 342 nm and 403 nm; λ_em_ = 378, 397 and 525 nm) at a concentration of 3 × 10^−6^ mol dm^−3^, at pH 7, sodium cacodylate buffer, I = 0.05 mol dm^−3^. Pyrene butyric acid has a stronger emission compared to compound **2** and was measured with a lower sensitivity instrumental setup.

Detailed analysis of the UV/Vis spectra (Figure 1 and Figure 2) showed the absorption maxima at ca. 400 nm, which can be assigned to the triarylborane unit, and only for compound **2**, absorption maxima at 332 and 347 nm corresponding to the pyrene moiety. Pyrene butyric acid (Pyr, Scheme 1) showed a very similar UV/Vis absorption spectrum to pyrene–triarylborane conjugate **2** (Figure 1 Top).

In fluorimetric experiments, λ = 342 nm was chosen as the excitation wavelength to excite the pyrene unit selectively, while the triarylborane chromophore of **1** and **2** was excited at 400 nm to emit at ca. 530 nm (Figure 1, Bottom).

There is a strong overlap between the pyrene emission centering at 375–450 nm (black line in Figure 2) and the UV/vis spectrum of the triarylborane (300–450 nm, pink line in Figure 2); with the spectral overlap integral [19,29] J = 1.54 × 10^14^ M^−1^ cm^−1^ (nm)^4^, therefore a principal requirement of FRET is fulfilled (energy transfer from the pyrene to the triarylborane). Indeed, upon excitation of **2** at 342 nm (black line in Figure 2), emissions at 378 and 397 nm agree well with the fluorescence of the pyrene (see the emission of pyrene butyric acid, green line in Figure 2), whereas the simultaneous appearance of the broad band at 525 nm can only be attributed to the FRET-caused fluorescence of triarylborane. The analysis showed that the ratio between 378 nm emission (λ_exc_ = 342 nm) and excitation spectrum (λ_em_ = 525 nm) could be used as a relative indicator of the efficiency of the FRET process here being estimated at less than 10%. More detailed analysis of the FRET efficiency in **2** by route of the average decay time of the pyrene detected at 340 nm, compared to the decay time of pyrene butyric acid (Pyr), according to Equations (S3) and (S4) in the Supplementary Information, yielding Φ_FRET_ of 0.023 (Table 2).

Quantum yields of fluorescence (Φ_f_) were measured in an aqueous medium by use of the fluorescence standards with optical properties closely matching the sample **1** and **2**: quinine sulphate in 0.5 M H_2_SO_4_ (Φ_f_ = 0.546) [30] and 9-cyanoantracene in MeOH (Φ_f_ = 0.80) [31]. The fluorescence quantum yields are generally low, as well as FRET efficiency (Table 2).

Singlet excited state lifetimes for **1** and **2** were measured by time-correlated single-photon counting (TC-SPC, Supplementary Information, Figures S5–S7 and Table S1). The samples were first excited at 340 nm, where dominantly pyrene absorbs light, and the emission was detected at 378 and 530 nm for **2** and only weak emission at 530 for **1** (due to small absorbance of triarylborane). Fluorescence decay was described as a sum of two or three exponentials giving lifetimes around 4 ns at 530 nm and ca. 120 ns at 378 nm, both values in good agreement with known data for triaryboranes [8] and pyrenes [19], respectively. When exciting the samples at 400 nm (exclusive triarylborane chromophore excitation) emission was detected at 530 nm providing lifetimes around 3 ns—in good agreement with previously published close analogues [8].

#### 2.2.2. Interactions with DNA and Proteins

DNA: In a previous study [8], it was demonstrated that the neutral triarylborane terminal alkyne A binds to the hydrophobic pocket of the protein BSA with high affinity and does not interact with ds-DNA. Nonetheless, we wanted to examine whether there were any interactions of the new compounds **1** and **2** with DNA, and calf thymus (ct)-DNA was chosen as a representative of typical B-helix structures with a balanced ratio of GC-(48%) and AT-(52%) base pairs. Again, preliminary thermal denaturation measurements [32] (Supplementary Information, Figure S8) and fluorescence tests by adding 5 and 20 equivalents of DNA (Supplementary Information, Figures S3 and S4) did not show any significant interaction with ds-DNA. Thus, **1** and **2** do not interact with nucleic acids.

**Figure 3:**
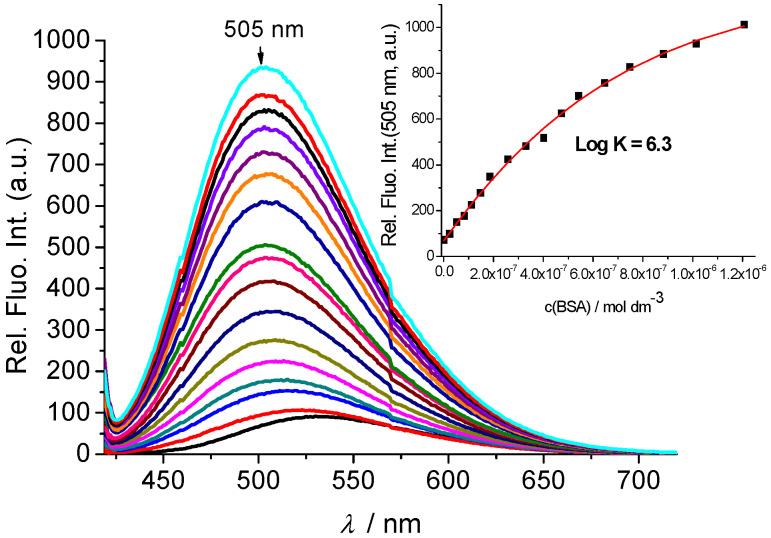
Fluorimetric titration of **1** (*c* = 5 × 10^−7^ mol dm^−3^; λ_exc_ = 400 nm) with **BSA** (bovine serum albumin). RIGHT: dependence of fluorescence at λ_max_ = 505 nm on c(BSA); the red line is non-linear least square fitting of experimental data for 1:1 = **1**/BSA stoichiometry. Done at pH 7, sodium cacodylate buffer, I = 0.05 mol dm^−3^.

Proteins: In our recent studies, both, cationic and neutral triarylborane conjugates showed strong interactions and corresponding fluorimetric recognition of the protein BSA [6,8], the most abundant protein in plasma. Analogously, the addition of BSA resulted in an emission increase of reference amino acid conjugate **1** (Figure 3), revealing a rather high binding constant (logKs = 6.3), in line with previously determined affinities of triarylborane analogue [8]. It should be stressed that the emission maximum of **1**/BSA complex was blue-shifted to 505 nm compared with that of free **1** (530 nm).

**Figure 4:**
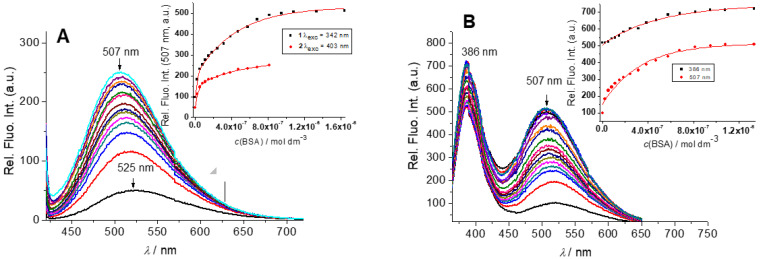
Fluorimetric titration of **2** (*c* = 5 × 10^−7^ mol dm^−^^3^) with BSA: (**A**) λ_exc_ = 403 nm; Inset: comparison of fluorescence spectra changes of **2** (*c* = 5 × 10^−7^ mol dm^−3^) at λ_max_ = 507 nm at different λ_exc_ (342 nm and 403 nm)—note that 342 nm excites only pyrene and nevertheless emission at 507 nm of triarylborane is observed; (**B**) λ_exc_ = 342 nm; Inset: dependence of fluorescence at λ_max_ = 386 nm and 507 nm on c(BSA); the red line is non-linear least-square fit to the experimental data for 1:1 = **2**/BSA stoichiometry. Done at pH 7, sodium cacodylate buffer, I = 0.05 mol dm^−3^. Note the significant hypsochromic shift (λ = −18 nm) of the **2**/BSA complex maximum in comparison to the emission maximum of **2**.

Titration of the triarylborane-pyrene conjugate **2** with BSA (Figure 4) resulted in a similar emission increase and similar binding constant (logK_s_ = 6.4) as obtained for reference compound **1**. It is noteworthy that excitation of **2** at the pyrene maximum (342 nm) and triarylborane maximum (403 nm) both gave the same titration profiles and binding affinities with BSA, indicating that both chromophores are similarly engaged in complex formation, at variance with previously studied cyanine–pyrene analogues, wherein pyrene interacted dominantly with BSA [33]. When only the pyrene chromophore was excited (λ_exc_ = 342 nm), upon binding to BSA, triarylborane emission at 525 nm was also observed, indicating that intramolecular FRET from pyrene to triarylborane also occurs inside the protein binding site. The strong increase of pyrene emission supported insertion of pyrene unit into the strongly hydrophobic environment [19], whereby the similar increase observed for both BSA and DPP III, suggests similar hydrophobic interactions.

**Figure 5:**
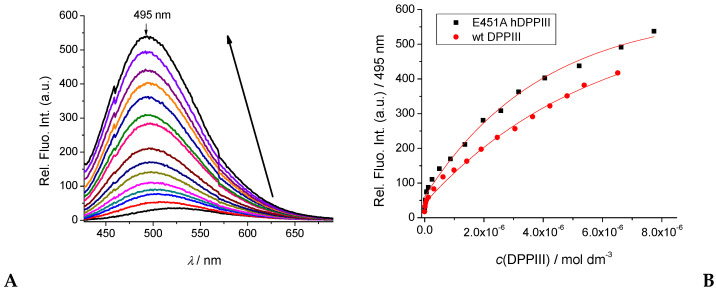
(**A**) fluorimetric titration of **1** (*c* = 5 × 10^−7^ mol dm^−3^; λ_exc_ = 400 nm) with hDPP III-E451A. (**B**) dependence of fluorescence at λ_max_ = 495 nm on c(hDPP III), E451A (black squares) and wt-type (red dots). Done in TRIS-HCl buffer, at pH 7.4 (I = 0.02 mol dm^−3^). Note the significant hypsochromic shift of the **1**/hDPP III-E451A complex in comparison to the emission of **1**.

Thus, establishing the strong interaction of **1** and **2** with BSA, a protein known to be a small-molecule carrier in the blood, the question arose whether **1** and **2** can interact with enzymes. For that purpose, we have chosen one type of peptidase enzyme, human DPP III (hDPP III), for which we previously reported several types of fluorescent markers [13,14], which also showed promising inhibitory activity of the enzyme action. As hDPP III could eventually cleave the peptide bond in **2**, we first performed fluorimetric titrations with mutant hDPP III-E451A, which due to its mutation at the active site, cannot cleave peptide bonds [11]. The emission increase obtained for **1** (Figure 5) and 2 (Supplementary Information, Figure S9), and binding constants (**1**: logKs = 6.6; **2**: logKs = 6.4) were very similar to those obtained for BSA, suggesting similar types of binding interactions.

**Figure 6:**
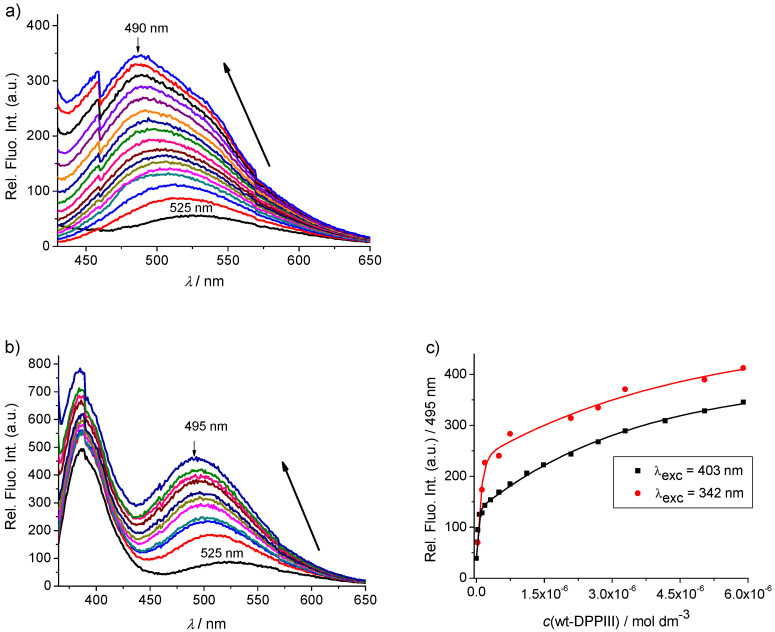
Fluorimetric titration of **2** (*c* = 5 × 10^−7^ mol dm^−3^) with wt-hDPP III. (**a**) λ_exc_ = 403 nm; (**b**) λ_exc_ = 342 nm; (**c**) dependence of fluorescence at λ_max_ = 495 nm on c(wt-DPP III) at λ_exc_ = 342 nm (red dots) and 403 nm (black squares), respectively. Done in TRIS-HCl buffer, at pH 7.4 (I = 0.02 mol dm^−3^). Note the significant hypsochromic shift of the **2**/wt-hDPP III complex in comparison to the emission of **2**.

Furthermore, we performed fluorimetric titrations with wild type (wt) hDPP III (Figure 5 bottom and Figure 6; and Supplementary Information, Figure S10) and obtained almost identical results as with the inactive mutant hDPP III-E451A.

Particularly for pyrene-triarylborane conjugate **2**, we determined that the emission of **2**/wt-hDPP III complex did not change with time, which could happen in the case of eventual enzymatic cleavage of the peptide bond in **2**. However, as in **2**, the fluorophores are not directly conjugated to peptide bond, the eventual enzymatic cleavage could result in minor emission changes. Thus, to check by an independent method for eventual enzymatic cleavage of the peptide bond, we performed ITC experiments under experimental conditions comparable to fluorimetric titrations (Supplementary Information, Figures S11 and S12). Namely, when the commercially available substrate for DPP III, Arg_2_-2NA, is cleaved by enzymatic action, the resulting heat change of the chemical reaction is at least two orders of magnitude larger in comparison to the enthalpy change caused by non-covalent binding of the substrate to inactive enzyme mutant E451A. We also performed a single injection ITC experiment under comparable conditions, which resulted in negligible enthalpy change, thus confirming that hDPP III does not cleave peptide bond in **2** on a fast timescale. However, these results do not exclude very slow enzymatic cleavage over a timescale of several hours or longer, which cannot be determined by ITC experiments.

### 2.3. hDPP III Kinetic Assays

As both the reference triarylborane–amino acid **1** and pyrene–triarylborane conjugate **2** bind with high affinities to wt-hDPP III, and neither of them are substrates for enzyme reactions, we studied their impact on the kinetics of wt-hDPP III enzymatic cleavage of the standard substrate Arg-Arg-2-naphthylamide (Arg_2_-2NA) by detecting the release of the product, 2-naphthylamine (λ_exc_ = 332 nm and λ_em_ = 420 nm) [34,35]. The results obtained are not affected by the minor impact of the intrinsic emission of **2**, which is several orders of magnitude weaker. We found that the reference triarylborane-amino acid **1** did not influence the enzymatic reaction of hDPP III (Supplementary Information, Figure S13, Table S3).

**Figure 7:**
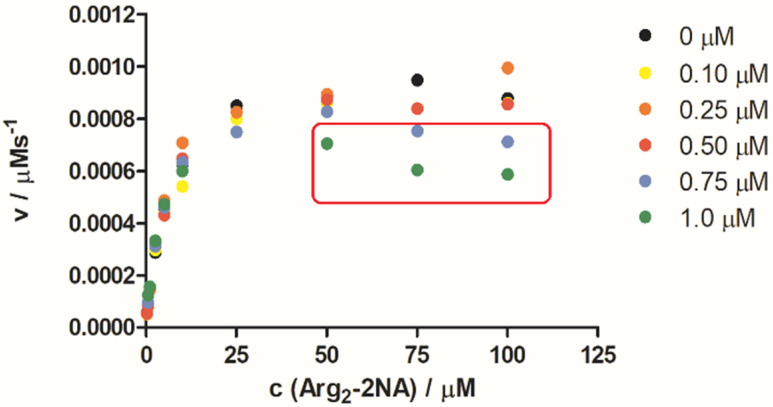
Unusual curves in Michaelis–Menten plots of DPP III in the presence of different concentrations of compound **2**.

All reaction profiles for c(**2**) < 0.25 μM follow the trend of common Michaelis–Menten plots (Figure 7), demonstrating undisturbed progress of enzymatic reaction; thus, **2** acts as a fluorescent marker for hDPP III with no impact on its enzymatic reaction. However, we observed that the addition of high concentrations of **2** (c > 0.25 μM) did influence the kinetic profile of the wt-hDPP III enzymatic reaction, but only at high c(Arg_2_-2NA) > 25 μM (Figure 7, red square), resulting in progressively lower reaction velocity, as seen most clearly at 0.75 and 1.0 μM **2**.

Such a decrease in a Michaelis–Menten plot is unusual, not resembling a typical inhibition curve, which should show a saturation plateau and not decrease. To us, it suggested the possibility of complex formation between the product of enzymatic reaction (2–naphthylamine) and compound **2**.

Therefore, to check whether **2** interacts with the substrate (Arg_2_-2NA) and/or product (naphthyl-amine), we performed fluorimetric titrations (Supplementary Information, Figures S14–S17). The results showed that conjugate **2** binds both substrate and product with considerable affinity (logK_s_ ~ 5). Thus, the formation of these complexes (**2**/substrate and **2**/product) impairs the enzymatic process at high concentrations of the dye c(**2**) > 0.25 μM) and c(Arg_2_-2NA) > 25 μM, resulting in an apparent decrease of the product fluorescence readout.

### 2.4. Molecular Modelling Studies

#### 2.4.1. Molecular Dynamics Simulations of the Complex of **2** and Substrate Arg_2_-2NA, **2**-Arg_2_-2NA, and Complex of **2** with Product **2**-Naphthylamine (2NA), **2**-2NA

**Figure 8:**
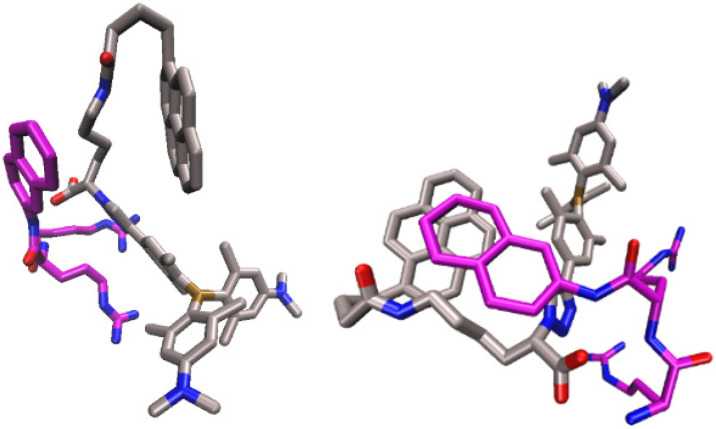
The representative structure of the complex between **2** (grey)- and Arg_2_-2NA (magenta), a member of the first class which contains 73% of all structures sampled during 200 ns of MD simulations in water (two orientations).

Intrigued by the unexpected stability of complexes of **2** with the small molecules Arg_2_-2NA and 2NA, we performed molecular dynamics simulations of the corresponding **2**-Arg_2_-2NA and **2**-2NA complexes. The results showed that these complexes could easily adopt several different orientations. To examine their populations and stabilities, we performed clustering. Structures sampled during 200 ns of MD simulations were clustered into 10 classes. In the case of the **2**-Arg_2_-2NA complexes, 73% of all generated structures were clustered into the first class, wherein the MMGBSA approximation of the free binding energy was calculated for the representative set of 30 structures close to the first class centroid is −35 + 3 kcal/mol. The most representative frames (the closest to the class centroid are displayed in Figure 8.

**Figure 9:**
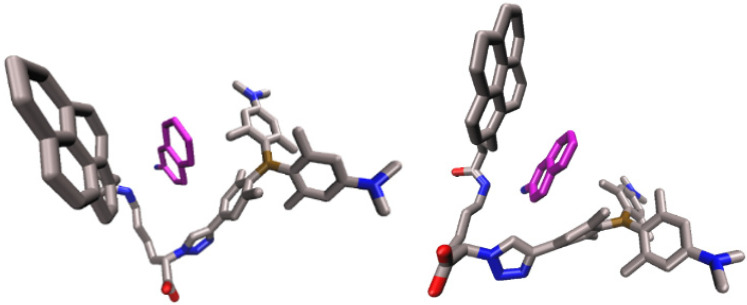
The representative structure of the complex between **2** (grey) and 2NA (magenta), a member of the first class which contains 37% of all structures sampled during 200 ns of MD simulations in water (two orientations).

Differently, the structures of the complex with the product, **2**-2NA, sampled during MD simulations vary more than those of **2**-Arg_2_-2NA structures, and the first class comprised only 37% of all structures. The MMGBSA approximation of the free binding energy calculated for the representative set of 30 structures close to the first class centroid is −8 + 2 kcal/mol. The most representative frame is displayed in Figure 9, exhibiting a sandwich-type form of a complex with the aromatic ligand inserted between two aromatic subunits of the receptor. The main driving force, in this case, resembles the classic supramolecular tweezer binding of aromatic substrates, similar to some supramolecular complexes we studied previously [36].

#### 2.4.2. Molecular Dynamic Simulations of the hDPP III-**2** Complex

**Figure 10:**
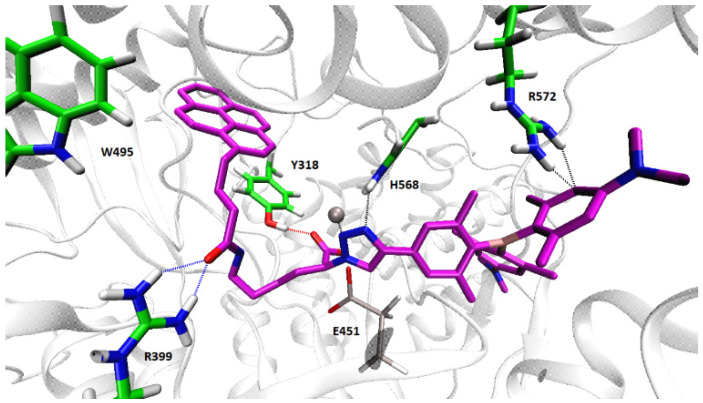
Representative structure of the hDPP III (closed)-**2** complex obtained during MD simulations in water with hydrogen bonds (H-bonds) displayed as dashed lines (blue and red are the strongest H-bonds, present during almost entire MD simulation, while hydrogen bonds shown in black are weaker, present during ca. 10−20% of the simulation time).

Four different initial structures of the hDPP III-**2** complex were prepared with **2** bound into the enzyme active site, two with the closed-form of hDPP III (well-defined hydrophobic pocket binding site) and two with partially open enzyme (binding site exposed to water, see Supplementary Information, Figures S18 and S19), and each of them was submitted to at least 100 ns of MD simulations. MMGBSA calculations combined with geometry and the hydrogen bond analysis revealed that complexes with the closed-form of hDPP III are more stable (Supplementary Information, Tables S4 and S5 and Figures S20 and S21) than the one with hDPP III in the partially open form. Apparently, intermolecular protein–ligand interactions are weaker in the latter form, as could also be seen from the population of hydrogen bonds during simulation (Supplemental Information Table S5). MMGBSA energies calculated on 2 ns intervals after every 8 ns (discrete separations) for the hDPP III-**2** complexes with the enzyme in its closed form and **2** bound in the binding mode1 are about −100 kcal/mol, while in the complex in which the enzyme binding site is more solvent-exposed, the MMGBSA energies are about −60 kcal/mol (see Supplemental Information Table S4). Complexes with **2** bound in the binding mode2 have MMGBSA energies of about −60 and −30 kcal/mol, respectively, (for hDPP III in its closed and semi-closed form). The most stable hDPP III-**2** complex is the one with hDPP III closed and ligand in the binding mode1 and the one with enzyme semi-closed and **2** in the binding mode2 with RMSD never exceeding 2 Å during MD simulations (Supplemental Information Figure S20). During all simulations, except for the hDPP III (semi-closed)-**2** (mode1) complex, **2** firmly interacts with the enzyme through several strong hydrogen bonds as well as through a series of van der Waals and electrostatic interactions (Supplemental Information Table S5) of which, in the case of hDPP III closed, the strongest is with the zinc ion Tyr318 and Arg399 as shown in Figure 10. The pyrene unit is accommodated in the hydrophobic region of the interdomain binding cleft and it makes a strong face-to-face interaction with the Tyr318 ring as well as a weak face-to-edge interaction with Trp495.

**Figure 11:**
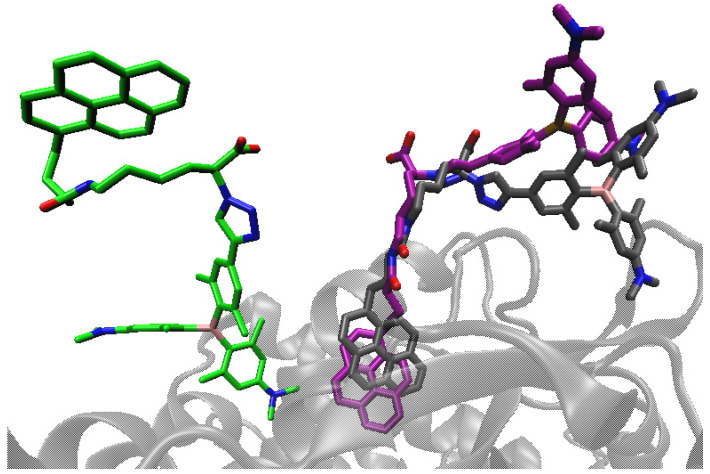
Structure of the hDPP III-**2** complex with **2** bound on the protein surface (light grey ribbon) after 5 (green), 150 (dark grey), and 210 (magenta) ns of MD simulations in water.

However, binding of **2** into the active site of DPP III and its interaction with catalytic amino acid residues would suggest a competitive binding mode, and the kinetic data suggested uncompetitive inhibition, so we also built a DPP III-**2** complex with **2** bound far from the enzyme active site and performed 210 ns of MD simulation. During the first 140 ns of MD simulation, **2** migrated over the protein surface and changed its initial conformation adopting a more stable binding mode (Figure 11). Although less favorable than the binding of **2** into the enzyme active site, the complex was stable during the following 90 ns with the MMGBSA energies below −30 kcal/mol (see Supplemental Information Table S6). In this position, **2** is stabilized with Thr419, Arg421, Lys423, and Lys439 (Supplemental Information Figure S23).

**Figure 12:**
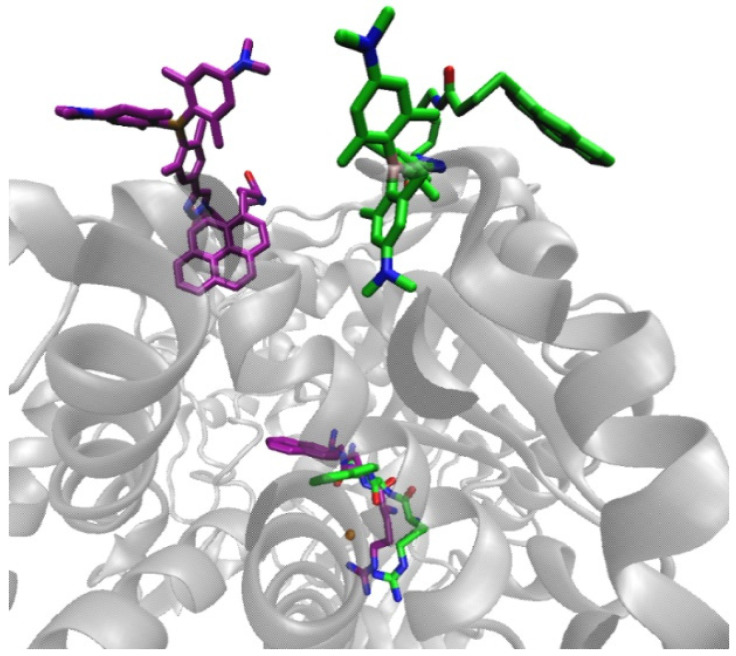
Superposition of two structures (I and II) of the hDPP III-Arg_2_-2NA-**2** complex in which Arg_2_-2NA is bound in the substrate-binding site and **2** is bound at the protein surface, obtained after 100 ns of MD simulations in water. For simplicity, only one protein structure, represented as grey ribbon, is shown, while structures of the small molecules, Arg_2_-2NA and **2** are colored magenta (structure I) and green (structure II), whereas substrate is multi-coloured in center, in the enzyme active site.

Further on, we built two complexes of DPP III with the Arg_2_-2NA into the active enzyme site and **2** bound at hDPP III surface and simulated each for 100 ns. The structure of the complexes obtained at the end of MD simulation are shown in Figure 12, and MMGBSA energies are given in Supplemental Information Table S7. According to the MMGBSA energies, binding of the hydrophobic pyrene into the protein with triaryl-borane water exposed is more favorable than burying one arm of the triaryl-borane into the protein and leaving pyrene water exposed.

The molecular modeling results showed that **2** can bind at the protein surface without hindering the binding of the substrate Arg2 2NA into the enzyme active site.

## 3. Materials and Methods

### 3.1. General Information

Solvents were distilled from appropriate drying agents shortly before use. All other reagents were used as obtained from Sigma Aldrich (St. Louis, MO, USA). TLC was carried out on TLC Silica gel 60 F254 plastic sheets and a preparative thick layer (2 mm) chromatography was done on Merck 60 F254 plates (Merck KGaA, Darmstadt, Germany). NMR spectra were recorded on Bruker AV600 and AV300 MHz spectrometers operating at 150.92 or 75.47 MHz for ^13^C and 600.13 or 300.13 MHz for ^1^H using DMSO-*d*_6_ as the solvent and internal reference. High-resolution mass spectra (HRMS) were obtained using a Thermo Fisher Scientific Exactive Plus Orbitrap MS System.

### 3.2. Synthesis

*Protected triarylborane-amino acid conjugate **C***: To a solution of alkyne **A** (68 mg, 0.16 mmol) in anhydrous DMF (2 mL), azide **B** (50 mg, 0.19 mmol), CuI (59.4 mg, 0.31 mmol), and DIPEA (42.8 µL, 0.47 mmol) were added and the reaction was stirred overnight. The solvent was removed under reduced pressure and the mixture of dichloromethane and methanol (20 mL) was added. The suspension was filtered through a small pad of celite to remove the catalyst and the filter cake was further washed with warm methanol. The volatiles were removed in vacuo and product **C** (94 mg, 85%) was isolated by preparative chromatography (EtOAc/MeOH/isoporopanol 7:3:1) as a yellow powder: R*_f_* = 0.45 (EtOAc/MeOH/isoporopanol 7:3:1); ^1^H-NMR (300 MHz, DMSO-*d*_6_) *δ*/ppm: 8.45 (s, 1H, triazole), 7.40 (s, 2H, 2 × CH), 6.72 (m, 1H, *J* = 5.5 Hz, NH), 6.30–6.29 (m, 4H, 4 × CH), 4.91 (br s, 1H, CH), 2.91–2.84 (m, 12H, 4 × NCH_3_), 2.15–1.89 (m, 20H, CH_2_ + 6 × CH_3_), 1.36–1.05 (m, 15H, 3 × CH_2_ + C(CH_3_)_3_); ^13^C-NMR (75 MHz, DMSO-*d*_6_) *δ*/ppm: 171.2 (Cq), 155.5 (Cq), 150.8 (Cq), 148.3 (Cq), 145.7 (Cq), 142.0 (Cq), 141.6 (Cq), 139.9 (Cq), 134.9 (Cq), 130.9 (Cq), 123.8 (CH), 120.4 (CH), 111.4 (CH), 111.3 (CH), 77.2 (C(CH_3_)_3_), 65.6 (CH), 40.1 (CH_3_), 39.9 (CH_3_), 39.8 (CH_3_), 39.5 (CH_3_), 32.4 (CH_2_), 31.3 (CH_2_), 29.0 (CH_2_), 28.2 (CH_3_), 23.5 (CH_3_), 23.2 (CH_3_), 23.1 (CH_2_), 22.5 (CH_3_); HRMS (APCI^-^): *m/z* found: 707.4480 [M − H]^−^; calcd. for [C_41_H_57_BN_6_O_4_-H]^−^ 707.4462 (|Δ| = 2.54 ppm).

*Triaryl-borane-amino acid conjugate***1**: Compound **C** (168 mg, 0.24 mmol) was dissolved in a 1:1 mixture of TFA/CH_2_Cl_2_ (10 mL) and stirred at room temperature for 3 days. After removal of the remaining TFA under reduced pressure, **1** (179 mg, 100%) was isolated as a yellow powder. ^1^H-NMR (600 MHz, DMSO-*d*_6_) *δ*/ppm: 8.68 (s, 1H, triazole), 7.73 (br.s., 3H, NH_3_), 7.42 (s, 2H, 2 × CH), 6.33–6.18 (m, 4H, 4 × CH), 5.41 (t, 1H, *J* = 7.5 Hz, CH), 2.92–2.72 (m, 12H, 4 × NCH_3_), 2.27–2.19 (m, 2H, CH_2_), 2.02–1.90 (m, 18H, 6 × CH_3_), 1.62–1.50 (m, 2H, CH_2_), 1.33–1.10 (m, 4H, 2 × CH_2_); ^13^C-NMR (151 MHz, DMSO-*d*_6_) *δ*/ppm: 170.2 (Cq), 158.2 (Cq), 157.9 (Cq), 150.7 (Cq), 148.8 (Cq), 146.5 (Cq), 142.1 (Cq), 141.6 (Cq), 140.0 (Cq), 135.3 (Cq), 130.3 (Cq), 123.9 (CH), 121.1 (CH), 111.6 (CH), 62.1 (CH), 40.1 (CH_3_), 39.9 (CH_3_), 38.5 (CH_2_), 30.4 (CH_2_), 26.3 (CH_2_), 23.6 (CH_3_), 23.1 (CH_3_), 22.5 (CH_3_), 22.4 (CH_2_), 18.5 (CH_3_); HRMS (APCI^+^): m/z found: 609.4092 [M]^+^; calcd. for [C_36_H_50_BN_6_O_2_]^+^ 609.4083 (|Δ| = 1.47 ppm).

*Triaryl-borane-amino acid-pyrene conjugate* **2**: Conjugate **1** (71 mg, 0.09 mmol) and 1-pyrenebutyric acid (25.9 mg, 0.09 mmol) were dissolved in anhydrous DMF (2 mL) under argon and HOBt (12.2 mg, 0.09 mmol), HBTU (34.1 mg, 0.09 mmol) and dry Et_3_N (50.1 µL, 0.36 mmol) were added. The reaction mixture was stirred at room temperature overnight. Product **2** (32 mg, 40%) was isolated by preparative chromatography (CH_2_Cl_2_/CH_3_OH 9:1) as a yellow powder: R*_f_* = 0.7 (CH_2_Cl_2_/MeOH, 9:1); ^1^H-NMR (600 MHz, DMSO-*d*_6_) *δ*/ppm: 8.67–7.77 (m, 11H, pyrene + triazole + NH), 7.46–7.43 (m, 2H, 2 × CH), 6.30–6.10 (m, 4H, 4 × CH), 5.23 (s, 1H, CH), 3.03–2.89 (m, 12H, 4 × NCH_3_), 2.16–1.82 (m, 26H, 6 × CH_3_ + 4 × CH_2_), 1.41 (br s., 2H, CH_2_), 1.23–1.04 (m, 4H, 2 × CH_2_); ^13^C NMR (151 MHz, DMSO-*d*_6_) *δ*/ppm: 172.4 (Cq), 168.0 (Cq), 151.1 (Cq), 151.0 (Cq), 150.9 (Cq), 149.1 (Cq), 146.6 (Cq), 142.9 (Cq), 140.3 (Cq), 136.6 (Cq), 135.2 (Cq), 131.0 (Cq), 130.6 (Cq), 129.4 (Cq), 128.3 (Cq), 127.6 (CH), 127.3 (CH), 126.6 (CH), 126.2 (CH), 125.0 (CH), 124.9 (CH), 124.4 (Cq), 124.3 (Cq), 124.1 (CH), 123.5 (CH), 120.9 (CH), 111.5 (CH), 63.2 (CH), 40.0 (CH_3_), 39.9 (CH_3_), 39.8 (CH_3_), 38.2 (CH_2_), 35.2 (CH_2_), 32.2 (CH_2_), 31.0 (CH_2_), 29.4 (CH_3_), 28.4 (CH_2_), 27.6 (CH_2_), 22.9 (CH_2_), 22.5 (CH_3_); HRMS (APCI^+^): *m/z* found: 879.5123 [M + H]^+^; calcd. for [C_56_H_63_BN_6_O_3_+H]^+^ 879.5127 (|Δ| = 0.45 ppm).

### 3.3. Spectroscopic Titrations

Calf thymus (ct)-DNA (Aldrich) was dissolved in sodium cacodylate buffer, I = 0.05 M, pH 7.0. The ct-DNA was additionally sonicated and filtered through a 0.45 μm filter to obtain mostly short (approximately 100 base pairs) rod-like B-helical DNA fragments.

Bovine Serum Albumin (BSA) (Sigma Aldrich) was dissolved in sodium cacodylate buffer, I = 0.05 M, pH 7.0, and its concentration was determined spectroscopically using a NanoDrop spectrophotometer at 280 nm using its molar extinction coefficient of 43,824 M^−1^ cm^−1^

UV-Vis spectra were recorded on a Varian Cary 100 Bio spectrometer in quartz cuvettes (1 cm). Fluorescence spectra were recorded on a Varian Cary Eclipse fluorimeter in quartz cuvettes (1 cm). Fluorimetric titrations were performed by adding portions of a protein stock solution into a solution of the compound, subsequently collecting emission spectrum, allowing the establishment of thermodynamic equilibrium for 120 s. After the end of the titration, the fluorescence spectra of dye/protein complexes remained constant for more than 1 h, proving that equilibrium was reached and no other processes (e.g., cleavage) are involved. Titration data were plotted in Origin 7.0 software, corrected for dilution (cumulative addition during titration was <5% of starting volume), and fitted to the first exponential equation, adequate for 1:1 stoichiometry of compound/protein complex. For all cases, good agreement between experimental and fitted data was observed, correlation coefficients being >0.99 for all calculated Ks values, thus supporting the dominant formation of a 1:1 stoichiometry complex.

### 3.4. Fluorescence Quantum Yields and Fluorescence Decays

The concentrations were adjusted to absorbances of less than 0.1 at the excitation wavelengths of 310, 320, 330 nm or 365, 375, and 385 nm. Fluorescence quantum yields were determined by comparison of the integral of the emission bands with the one of quinine sulfate in 0.05 M H_2_SO_4_ (Φ_f_ = 0.546) [30] or 9-cyanoantracene in MeOH (Φ_f_ = 0.8) [31]. One fluorescence measurement was performed by the excitation of the sample at three different wavelengths and the average value was calculated (Supplemental Information Equation (S1)). Before the measurements, the solutions were purged with Ar for 15 min. The measurements were performed at rt (25 °C) in sodium cacodylate buffer, pH 7, I = 0.05 mol dm^−3^. Fluorescence decays were measured by the TC-SPC method on an Edinburgh FS5 spectrometer equipped with pulsed LEDs at 340 nm and 400 nm. Fluorescence signals were monitored over 1023 channels with a time increment of ≈20 ps/channel. The decays were collected until they reached 500 counts in the peak channel. A suspension of silica gel in H_2_O was used as a scattering solution to obtain instrument response function (IRF). The histograms were analyzed by a nonlinear least-squares deconvolution method using Equation (S2) in the Supplemental Information. The quality of the fit was judged by the reduced χ2 being close to unity and the random distribution of the weighted residuals.

### 3.5. Purification of Human DPP III

Recombinant human DPP III was obtained by heterologous expression in *Escherichia coli* and purification as previously described [37]. Briefly, the gene for DPP III with a C-terminal His-tag was expressed in E. coli from a pET21b plasmid using 0.5 mM isopropyl β-d-1-thiogalactopyranoside (IPTG). Bacterial pellets were resuspended in a 50 mM Tris-HCl buffer pH = 8.0 with 300 mM NaCl and 10 mM imidazole and lysed using 1 mg/mL lysozyme. The lysate was cleared by ultracentrifugation at 15,000× *g* for 45 min and by filtration through a 0.45 μm filter. Protein purification was performed on a Ni-NTA column, with washing and elution using buffers with 20 mM and 300 mM imidazole, respectively. Fractions were run on a gel [38], pooled, desalted, and stored at −80 °C.

### 3.6. DPP III Kinetic Assay

The enzymatic activity of wild-type human DPP III was determined by a standard fluorimetric assay at 25 °C with Arg_2_-2-naphthylamide as the substrate, where the fluorescent 2-naphtyplamine is released due to enzymatic cleavage and measured (excitation at 332 nm and emission at 420 nm). The concentration of human DPP III in the reaction mixture was 0.1 nM and the substrate concentration (Arg_2_-2NA) was varied from 0.25 to 100 μM. Compound **1** was added to the enzyme reactions at concentrations of 1 and 3 μM. The effect of compound **2** was measured at 0.1, 0.25, 0.5, 0.75, and 1.0 μM concentrations. Because of fluorescence of **2** at 420 nm, baseline fluorescence was measured for all reactions before the addition of the enzyme. All measurements were repeated in duplicate or triplicate. Data were analyzed using GraphPad Prism5 software (GraphPad, San Diego, CA, USA) according to the Michaelis–Menten model nonlinear regression.

### 3.7. Isothermal Titration Calorimetry (ITC)

To study the interaction of DPP III with our compounds by ITC, a MicroCal VP-ITC microcalorimeter was used at pH 7.4 in 20 mM Tris-HCl buffer. Origin 7.0 software, supplied by the manufacturer, was used for data analysis. The reference cell was filled with ultrapure water. Typically, titrations were performed such that one aliquot of 2 µL and 29 aliquots of 10 µL of **2** (*c* = 1 × 10^−4^ M) were injected from rotating syringe (112–270 rpm) into the isothermal cell, equilibrated at 25.0 °C, containing 1421 mL of hDPP III mutant E451A (*c* = 5–10 µM). The spacing between each injection was in the range of 300–600 s, and the initial delay before the first injection was in the range of 600–3000 s. All solutions used for ITC experiments were degassed before use under vacuum (0.64 bar, 10 min) to eliminate air bubbles. Microcalorimetric experiments directly gave three parameters: reaction enthalpy change (Δ_r_H), binding constant (log K_a_), and stoichiometry (N). The value of Δ_r_G was calculated from the binding constant (Δ_r_G = −RT ln K) and the reaction entropy change was calculated from the binding enthalpy and Gibbs free energy (Δ_r_S = Δ_r_H − Δ_r_G)/T).

### 3.8. Molecular Simulations

Parameterisation, energy minimization, and molecular dynamics (MD) simulations of the complexes between compound **2** and DPP III, as well as between **2** and the preferred synthetic substrate of DPP III Arg-Arg-2-naphthylamide (RR-2-NA), and the product of its hydrolysis 2NA were performed using the AMBER16 suite of programs [39]. The solutes were prepared using the AMBER16 utility program tLeap wherein the small molecules and DPP III were parametrized within general AMBER gaff [40] and ff14SB [41] force fields, respectively, wherein hybrid bonded-non bonded parameters were used for the zinc ion [42] (for details of the parametrization procedure, see our previous work [6,13]).

Initial **2**-Arg2 2NA and **2**-2NA complexes were prepared in PyMOL (The PyMOL Molecular Graphics System, Version 1.7 SchroÈdinger, LLC, New York, NY, USA), while the protein complexes with **2**, DPP III-**2** were prepared using the DPP III-tynorphine complex (pdb code: 3T6B) as a template.

The complexes were solvated in the truncated octahedron box filled with TIP3P water molecules [43] whereas the chloride ions were added to achieve electroneutrality of the **2**-Arg2 2NA and **2**-2NA complexes, and the sodium ions were added to achieve electroneutrality of DPP III-**2** and hDPP III-RRNA-**2** complexes. The solvated complexes were geometry optimized by using steepest descent and conjugate gradient methods (altogether 7000 steps and 13,500 steps, between small molecules and enzyme, respectively) and equilibrated for 100 ps and 200 ps (between small molecules and enzyme, respectively) with a time step of 1 fs. During the first stage of equilibration (30 ps), the temperature was linearly increased from 0 to 300 K and the volume was held constant. In the second stage (NPT ensemble with T and P about 300 K and 1 atm, respectively), the solution density was optimized. The equilibrated complexes were subjected to productive molecular dynamics simulation using NPT conditions and a time step of 2 fs. The temperature was held constant using a Langevin thermostat [44] with a collision frequency of 0.2 ps. The pressure was regulated by a Berendsen barostat [45]. The simulations were performed using periodic boundary conditions (PBC). The particle mesh Ewald (PME) method was used for the calculation of the long-range electrostatic interactions. The complexes between small molecules, **2**-Arg2 2NA and **2**-2NA complexes were simulated for 200 ns with the cut-off distance of 8 Å. In contrast, the cut-off value of 11 Å was used during simulations of DPP III-**2** complex and the simulation time was 100 ns for each of three different **2** orientations. The two low-energy complexes were additionally simulated for 70 ns. Minimization was performed by the program sander.MPI and equilibration and MD simulations by pmemd.cuda.MPI.

The binding free energies between **2** and Arg2 2NA and 2NA as well as for **2** binding to DPP III were evaluated using MM-GBSA calculations, utilizing the GB model of Onufriev et al. [46]. Geometry and the hydrogen bond population were analyzed by the CPPTRAJ module of the AmberTools program package [47]. Complexes between and substrate, Arg2 2NA, as well as with product 2NA were clustered using the “hieragglo” clustering algorithm. Clustering was based on RMSD calculated for the pair-wise distance between all atoms except hydrogens. The number of clusters was 10 and sieve 4 was used (clustering was performed for every fourth frame sampled during MD simulations). Radii of gyration, RMSF (root mean square fluctuation), and RMSD (root mean square deviation) values for the enzyme were calculated for the backbone atoms and averaged over atoms of a residue, while RMSD values of the ligand (**2**) were calculated for all atoms except hydrogens.

## 4. Conclusions

We report the facile synthesis of novel triarylborane dyes, prepared by attaching a triarylborane chromophore to an amino acid side chain by a “click CuAAC” reaction (**1**) and further conjugation of this amino acid with pyrene (**2**), the latter tandem dye forming an efficient pyrene-triarylborane FRET pair.

In contrast to previous cationic triarylboranes [6,8], the novel neutral dyes did not interact with DNA, but only with proteins. Both the reference triarylborane-amino acid **1** and triarylborane–pyrene conjugate **2** bind to BSA and the DPP III enzyme with high affinity, exhibiting a strong (up to 100-fold) fluorescence increase, whereby the pyrene conjugate additionally exhibited efficient FRET within a protein binding site. Furthermore, the triarylborane dyes, upon binding to the DPP III enzyme, did not impair its enzymatic affinity under a wide range of experimental conditions, thus being the first non-covalent markers for DPP III, which are also applicable during enzymatic reactions.

Further development of these fluorescent probes targeting DPP III is planned in two directions. The first one would be taking advantage of the synthetic adaptability of triarylborane-amino acid **1**, as well as pyrene conjugate **2**, by equipping them with potential DPP III inhibitors (e.g., the guanidiniocarbonylpyrrole unit which we used before [13,14]) to achieve novel, highly active and emissive enzyme inhibitors. Another application of **1**, **2** would be using them as non-covalent and non-inhibitive fluorescent markers for localization of DPP III in complex systems during studies of enzymatic activity. Of course, such systems should not contain other proteins (**1**, **2** bind BSA as well) but could contain a y-type of DNA/RNA or small molecules. For example, numerous enzymes are used in various solvents for highly selective synthesis or analysis, and a systematic check of enzyme concentration at any point of the process is of great importance.

## Figures and Tables

**Table 1:** UV/Vis absorption data ^a^ for **1** and **2**.

	λ_max_/nm	ε × 10^3^/mmol^−1^ cm^2^
**1**	256	19.7 ± 0.2
	310	7.7 ± 0.1
	400	11.3 ± 0.1
**2**	278	26.4 ± 0.6
	332	15.4 ± 0.6
	347	17.9 ± 0.6
	403	11.2 ± 0.6

^a^ A stock solution of **1** was prepared in water (about *c* = 1 × 10^−3^ mol dm^−3^). A stock solution of **2** was prepared in DMSO (ca. *c* = 1 × 10^−3^ mol dm^−3^).

**Table 2:** Quantum yields of fluorescence Φ_f_^a^, and Φ_f_^b^, and efficiency of FRET (Φ_FRET_) ^c^.

	Φ_f_^a^	Φ_f_^b^	Φ_FRET_^c^
**1**	0.011 ± 0.001	0.024 ± 0.001	
**2**	0.028 ± 0.002	0.008 ± 0.0001	0.023

^a^ Fluorescence quantum yield estimated using quinine sulfate as a standard. ^b^ Fluorescence quantum yield estimated using 9-cyanoantracene as a standard. The fluorescence spectra were measured by exciting at 310, 320, and 330 nm when using quinine sulfate, and 365, 375, and 385 nm when using 9-cyanoantracene as a standard. The average values of the quantum yields (Supplementary Information Equation (S1)) were calculated. The measurements were performed three times and the average values are reported. The associated errors correspond to the maximum absolute deviation. ^c^ The efficiency of the Förster resonance energy transfer (FRET) was determined by shortening of the average decay time of the pyrene detected at 340 nm, compared to the decay time of pyrene butyric acid (Pyr), according to Equations (S3) and (S4) in the Supplementary Information.

## Data Availability

The data presented in this study are available on request from the corresponding author.

## Supplementary Materials

Supplementary Materials are available online, Additional characterization data of novel molecules **1** and **2**, additional DNA/RNA binding data.

## Sample Availability

Samples of the compounds are not available from the authors.

## References

[1] Griesbeck S., Michail E., Wang C., Ogasawara H., Lorenzen S., Gerstner L., Zang T., Nitsch J., Sato Y., Bertermann R. (2019). Tuning the π-bridge of quadrupolar triarylborane chromophores for one- and two-photon excited fluorescence imaging of lysosomes in live cells. Chem. Sci..

[2] Ji L., Griesbeck S., Marder T.B. (2017). Recent developments in and perspectives on three-coordinate boron materials: A bright future. Chem. Sci..

[3] Griesbeck S., Michail E., Rauch F., Ogasawara H., Wang C., Sato Y., Edkins R.M., Zhang Z., Taki M., Lambert C. (2019). The Effect of Branching on the One- and Two-Photon Absorption, Cell Viability, and Localization of Cationic Triarylborane Chromophores with Dipolar versus Octupolar Charge Distributions for Cellular Imaging. Chem. Eur. J..

[4] Griesbeck S., Ferger M., Czernetzi C., Wang C., Bertermann R., Friedrich A., Haehnel M., Sieh D., Taki M., Yamaguchi S. (2019). Optimization of Aqueous Stability versus π-Conjugation in Tetracationic Bis(triarylborane) Chromophores: Applications in Live-Cell Fluorescence Imaging. Chem. Eur. J..

[5] Griesbeck S., Zhang Z., Gutmann M., Lühmann T., Edkins R., Clermont G., Lazar A.N., Haehnel M., Edkins K., Eichhorn A. (2016). Water-Soluble Triarylborane Chromophores for One- and Two-Photon Excited Fluorescence Imaging of Mitochondria in Cells. Chem. Eur. J..

[6] Ban Ž., Griesbeck S., Tomić S., Nitsch J., Marder T.B., Piantanida I. (2020). A Quadrupolar Bis-Triarylborane Chromophore as a Fluorimetric and Chirooptic Probe for Simultaneous and Selective Sensing of DNA, RNA and Proteins. Chem. Eur. J..

[7] Varshney A., Sen P., Ahmad E., Rehan M., Subbarao N., Khan R.H. (2010). Ligand binding strategies of human serum albumin: How can the cargo be utilized?. Chirality.

[8] Amini H., Ban Ž., Ferger M., Lorenzen S., Rauch F., Friedrich A., Crnolatac I., Kenđel A., Miljanić S., Piantanida I. (2020). Tetracationic Bis-Triarylborane 1,3-Butadiyne as a Combined Fluorimetric and Raman Probe for Simultaneous and Selective Sensing of Various DNA, RNA, and Proteins. Chem. Eur. J..

[9] Ferger M., Ban Ž., Krošl I., Tomić S., Dietrich L., Lorenzen S., Rauch F., Sieh D., Friedrich A., Griesbeck S. (2021). Bis(phenylethynyl)arene Linkers in Tetracationic Bis-triarylborane Chromophores Control Fluorimetric and Raman Sensing of Various DNAs and RNAs. Chem. Eur. J..

[10] Rawlings N., Waller M., Barrett A.J., Bateman A. (2014). MEROPS: The database of proteolytic enzymes, their substrates and inhibitors. Nucleic Acids Res..

[11] Agić D., Brkić H., Tomić S., Karačić Z., Špoljarević M., Lisjak M., Bešlo D., Abramić M. (2017). Validation of flavonoids as potential dipeptidyl peptidase III inhibitors: Experimental and computational approach. Chem. Biol. Drug Des..

[12] Matovina M., Agić D., Abramić M., Matić S., Karačić Z., Tomić S. (2017). New findings about human dipeptidyl peptidase III based on mutations found in cancer. RSC Adv..

[13] Ćehić M., Sajko J.S., Karačić Z., Piotrowski P., Šmidlehner T., Jerić I., Schmuck C., Piantanida I., Tomić S. (2020). The guanidiniocarbonylpyrrole–fluorophore conjugates as theragnostic tools for dipeptidyl peptidase III monitoring and inhibition. J. Biomol. Struct. Dyn..

[14] Suć J., Tumir L.-M., Glavaš-Obrovac L., Jukić M., Piantanida I., Jerić I. (2016). The impact of α-hydrazino acids embedded in short fluorescent peptides on peptide interactions with DNA and RNA. Org. Biomol. Chem..

[15] Šafranko Ž.M., Sobočanec S., Šarić A., Jajčanin-Jozić N., Krsnik Z., Aralica G., Balog T., Abramić M. (2015). The effect of 17β-estradiol on the expression of dipeptidyl peptidase III and heme oxygenase 1 in liver of CBA/H mice. J. Endocrinol. Investig..

[16] Prajapati S.C., Chauhan S.S. (2011). Dipeptidyl peptidase III: A multifaceted oligopeptide N-end cutter. FEBS J..

[17] Hast B.E., Goldfarb D., Mulvaney K.M., Hast M.A., Siesser P.F., Yan F., Hayes D.N., Major M.B. (2013). Proteomic Analysis of Ubiquitin Ligase KEAP1 Reveals Associated Proteins That Inhibit NRF2 Ubiquitination. Cancer Res..

[18] Matić S., Kekez I., Tomin M., Bogár F., Šupljika F., Kazazić S., Hanić M., Jha S., Brkić H., Bourgeois B. (2020). Binding of dipeptidyl peptidase III to the oxidative stress cell sensor Kelch-like ECH-associated protein 1 is a two-step process. J. Biomol. Struct. Dyn..

[19] Lakowicz J.R. (2006). Principles of Fluorescence Spectroscopy.

[20] Preus S., Wilhelmsson L.M. (2012). Advances in Quantitative FRET-Based Methods for Studying Nucleic Acids. ChemBioChem.

[21] König I., Zarrine-Afsar A., Aznauryan M., Soranno A., Wunderlich B., Dingfelder F., Stüber J., Plückthun A., Nettels D., Schuler B. (2015). Single-molecule spectroscopy of protein conformational dynamics in live eukaryotic cells. Nat. Methods.

[22] Tornøe C.W., Christensen C., Meldal M. (2002). Peptidotriazoles on Solid Phase: [1,2,3]-Triazoles by Regiospecific Copper(I)-Catalyzed 1,3-Dipolar Cycloadditions of Terminal Alkynes to Azides. J. Org. Chem..

[23] Rostovtsev V.V., Green L.G., Fokin V.V., Sharples K.B. (2002). A Stepwise Huisgen Cycloaddition Process: Cop-per(I)-Catalyzed Regioselective “Ligation” of Azides and Terminal Alkynes. Angew. Chem. Int. Ed..

[24] Altimari J.M., Niranjan B., Risbridger G.P., Schweiker S.S., Lohning A.E., Henderson L.C. (2014). Synthesis and preliminary investigations into novel 1,2,3-triazole-derived androgen receptor antagonists inspired by bicalutamide. Bioorg. Med. Chem. Lett..

[25] Vatmurge N.S., Hazra B.G., Pore V.S., Shirazi F., Chavan P.S., Deshpande M.V. (2008). Synthesis and antimicrobial activity of β-lactam–bile acid conjugates linked via triazole. Bioorg. Med. Chem. Lett..

[26] Whiting M., Tripp J.C., Lin Y.-C., Lindstrom W., Olson A.J., Elder J.H., Sharpless K.B., Fokin V.V. (2006). Rapid Discovery and Structure−Activity Profiling of Novel Inhibitors of Human Immunodeficiency Virus Type 1 Protease Enabled by the Copper(I)-Catalyzed Synthesis of 1,2,3-Triazoles and Their Further Functionalization. J. Med. Chem..

[27] Ban Ž., Matić J., Žinić B., Füchtbauer A.F., Wilhelmsson L.M., Piantanida I. (2020). Flexibility and Preorganization of Fluorescent Nucleobase-Pyrene Conjugates Control DNA and RNA Recognition. Molecules.

[28] Dourtoglou V., Gross B., Lambropoulou V., Zioudrou C. (1984). *O*-Benzotriazolyl-*N*,*N*,*N*′,*N*′-tetramethyluronium Hexafluorophosphate as Coupling Reagent for the Synthesis of Peptides of Biological Interest. Synthesis.

[29] FluorTools A|e—UV-Vis-IR Spectral Software 1.2. www.fluortools.com.

[30] Montalti M., Credi A., Prodi L., Gandolfi M.T. (2006). Handbook of Photochemistry.

[31] Boens N., Qin W., Basaric N., Hofkens J., Ameloot M., Pouget J., Lefèvre J.-P., Valeur B., Gratton E., Vandeven M. (2007). Fluorescence Lifetime Standards for Time and Frequency Domain Fluorescence Spectroscopy. Anal. Chem..

[32] Mergny J.-L., Lacroix L. (2003). Analysis of Thermal Melting Curves. Oligonucleotides.

[33] Šmidlehner T., Badovinac M., Piantanida I. (2018). Pyrene–cyanine conjugates as multipurpose fluorescent probes for non-covalent recognition of ds-DNA, RNA and proteins. New J. Chem..

[34] Abramić M., Šimaga S., Osmak M., Cicin-Sain L., Vukelić B., Vlahoviček K., Dolovčak L. (2004). Highly reactive cysteine residues are part of the substrate binding site of mammalian dipeptidyl peptidases III. Int. J. Biochem. Cell Biol..

[35] Kumar P., Reithofer V., Reisinger M., Wallner S., Pavkov-Keller T., Macheroux P., Gruber K. (2016). Substrate complexes of human dipeptidyl peptidase III reveal the mechanism of enzyme inhibition. Sci. Rep..

[36] Piantanida I., Palm B.S., Cudic P., Zinic M., Schneider H.-J. (2001). Phenanthridinium cyclobisintercalands. Fluorescence sensing of AMP and selective binding to single-stranded nucleic acids. Tetrahedron Lett..

[37] Špoljarić J., Salopek-Sondi B., Makarević J., Vukelić B., Agić D., Šimaga Š., Jajčanin-Jozić N., Abramić M. (2009). Absolutely conserved tryptophan in M49 family of peptidases contributes to catalysis and binding of competitive inhibitors. Bioorg. Chem..

[38] Laemmli U.K. (1970). Cleavage of structural proteins during the assembly of the head of bacteriophage T4. Nature.

[39] Case D., Betz R., Cerutti D., Cheatham T.E., Darden T., Duke R.E., Giese T., Gohlke H., Goetz A.W., Homeyer N. (2016). AMBER 2016.

[40] Wang J., Wolf R.M., Caldwell J.W., Kollman P.A., Case D.A. (2004). Development and testing of a general amber force field. J. Comput. Chem..

[41] Maier J.A., Martinez C., Kasavajhala K., Wickstrom L., Hauser K., Simmerling C. (2015). ff14SB: Improving the Accuracy of Protein Side Chain and Backbone Parameters from ff99SB. J. Chem. Theory Comput..

[42] Tomić A., Horvat G., Ramek M., Agić D., Brkić H., Tomić S. (2019). New Zinc Ion Parameters Suitable for Classical MD Simulations of Zinc Metallopeptidases. J. Chem. Inf. Model..

[43] Jorgensen W.L., Chandrasekhar J., Madura J.D., Impey R.W., Klein M.L. (1983). Comparison of simple potential functions for simulating liquid water. J. Chem. Phys..

[44] Loncharich R.J., Brooks B.R., Pastor R.W. (1992). Langevin dynamics of peptides: The frictional dependence of isomerization rates of *N*-acetylalanyl-*N*′-methylamide. Biopolymers.

[45] Berendsen H.J.C., Postma J.P.M., Van Gunsteren W.F., DiNola A., Haak J.R. (1984). Molecular dynamics with coupling to an external bath. J. Chem. Phys..

[46] Onufriev A., Bashford D., Case D.A. (2004). Exploring protein native states and large-scale conformational changes with a modified generalized born model. Proteins.

[47] Roe D.R., Cheatham T.E. (2013). PTRAJ and CPPTRAJ: Software for processing and analysis of molecular dynamics trajectory data. J. Chem. Theory Comput..

