# Supplementary material for: Triarylborane Dyes as a Novel Non-Covalent and Non-Inhibitive Fluorimetric Markers for DPP III Enzyme
Source: Molecules. 2021 Aug 9;26(16):4816. doi: 10.3390/molecules26164816 (PMC8398983; doi:10.3390/molecules26164816)
Supplement: Supplementary file 1 [file molecules-26-04816-s001.zip › molecules-1305038-supplementary.pdf]

Supporting Information

Triarylborane dyes as a novel non-covalent and non-inhibitive fluorimetric markers for DPP III enzyme

Željka Ban,[a] Zrinka Karačić,[a] Sanja Tomić,[a]* Hashem Amini,[b] Todd B. Marder,[b] Ivo Piantanida[a]*

[a] Division of Organic Chemistry & Biochemistry, Ruđer Bošković Institute, Zagreb, Croatia

[b] Institut für Anorganische Chemie and Institute for Sustainable Chemistry & Catalysis with Boron Julius-Maximilians-Universität Würzburg, 97074 Würzburg Germany

Contents

Photophysical measurements……………………………..……........………………….....S2

Thermal denaturation experiments…………………………………………………….S8

Study of interactions with proteins…………….……………..……………………........S9

DPP III kinetic assay ………………………………………………….………....S11

Molecular simulations………………………………………………………………….S14

NMR and HRMS Spectra……………………………………...…………………….S19

References……………………………………………………………………………S24

Photophysical measurements in buffered solution (sodium cacodylate pH=7)

UV/Vis data:

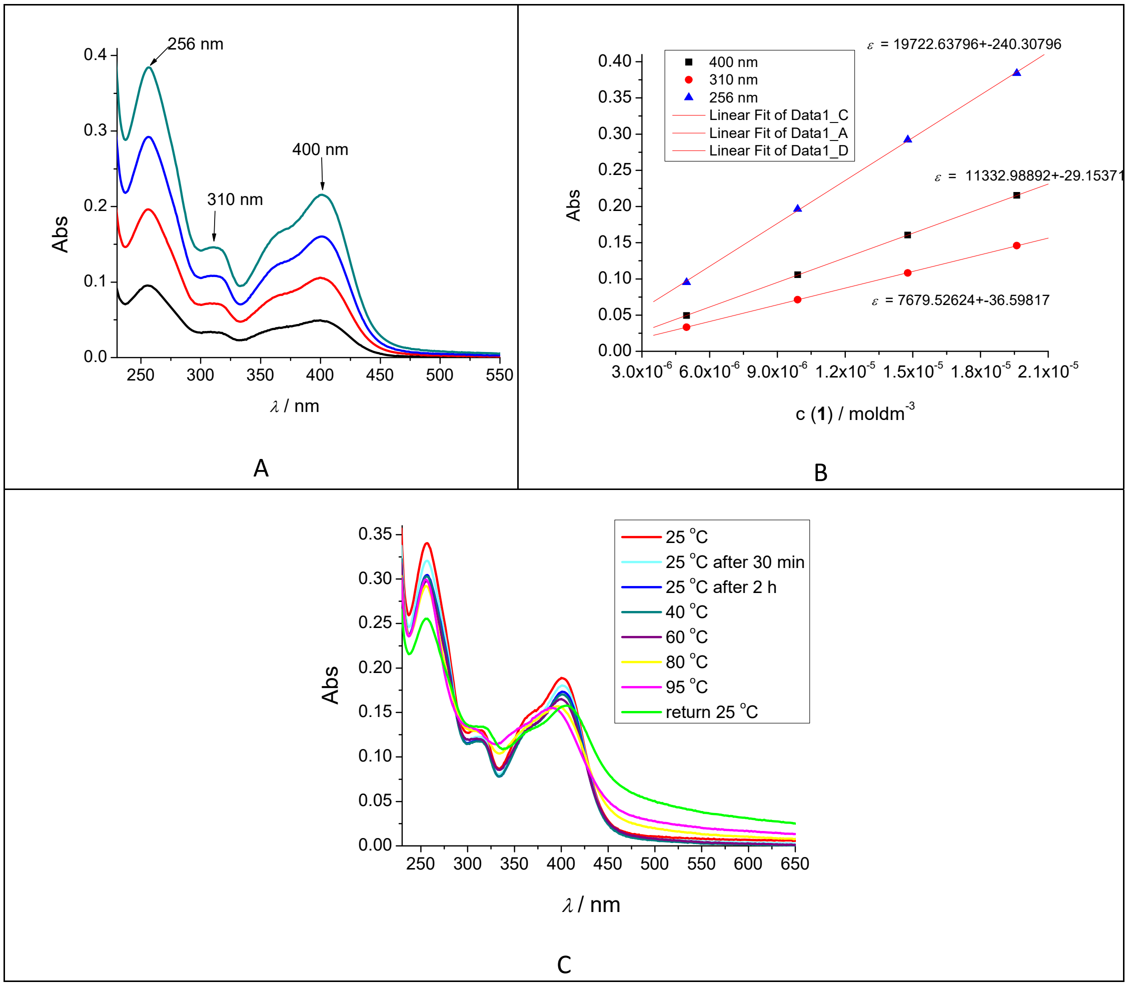

Figure S1. A: UV/Vis spectra of **1**, c = 5×10^{-6} - 2×10^{-5} mol dm^{-3}; B: linear dependence (—) of the absorbance at 256 (▲), 310 (●) and 400 nm (■) on the **1** concentration; C: time and temperature dependence (T = 25°C – 95 °C) of UV/Vis spectra (c = 2×10^{-5} mol dm^{-3}). Performed in Na-cacodylate buffer, pH = 7.0, I = 0.05 mol dm^{-3}.

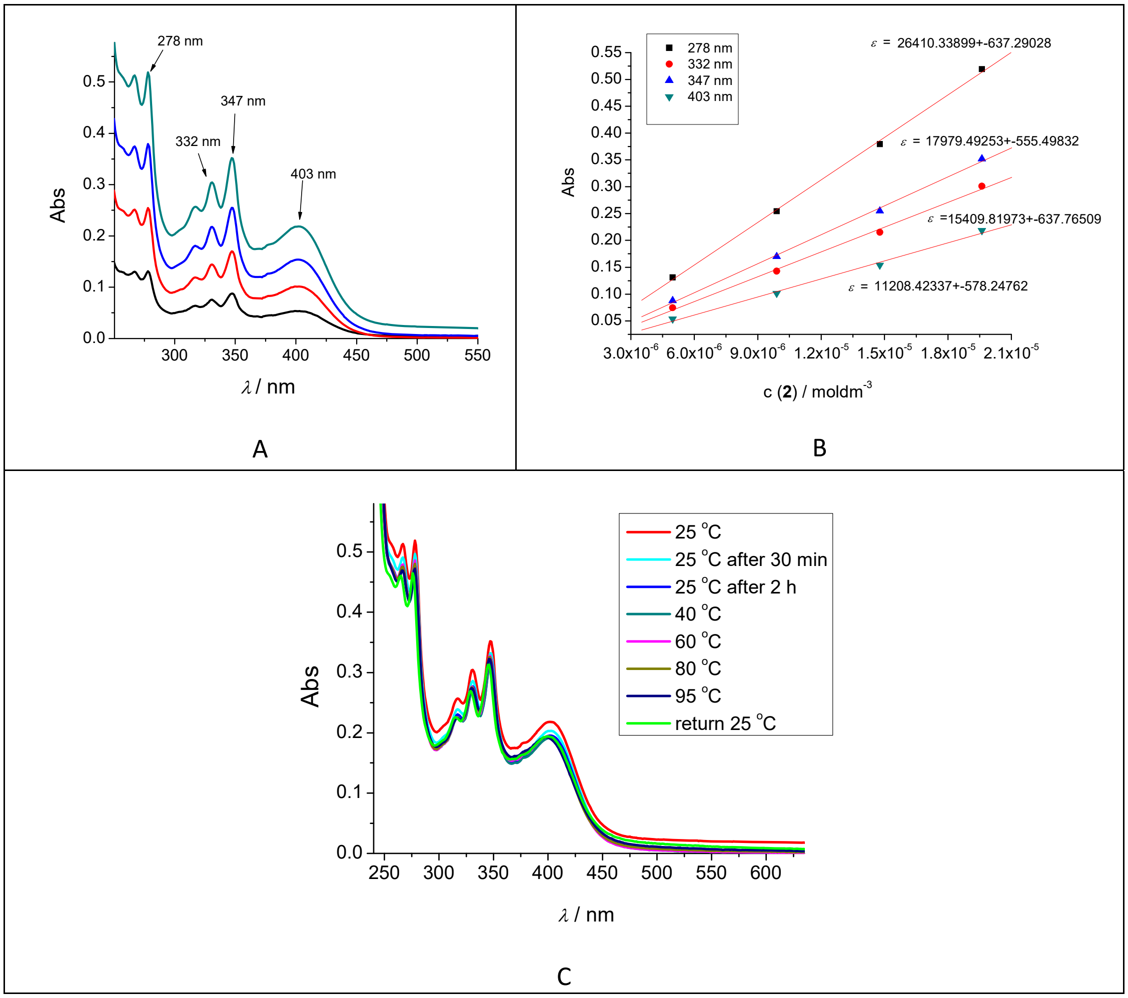

Figure S2. A: UV/Vis spectra of **2**, c = 5×10^{-6} - 2×10^{-5} mol dm^{-3}; B: linear dependence (—) of the absorbance at 278 nm (■), 332 (●), 347 (▲), and 403 nm (▲) on the **2** concentration; C: time and temperature dependence (T = 25°C – 95 °C) of UV/Vis spectra (c = 2×10^{-5} mol dm^{-3}). Performed in Na-cacodylate buffer, pH = 7.0, I = 0.05 mol dm^{-3}.

Fluorimetric spectra:

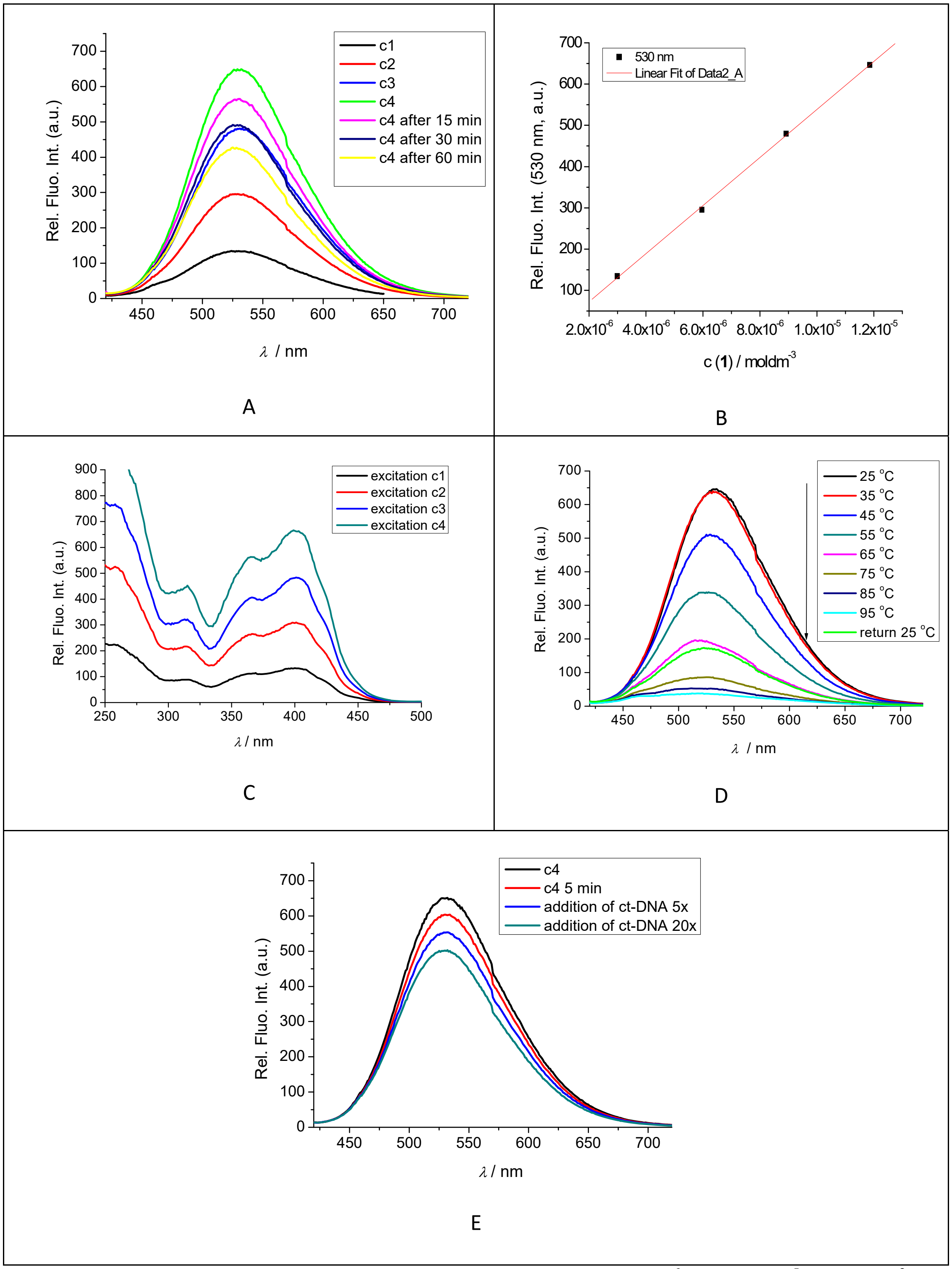

Figure S3. A: Emission spectra of **1** (λ_{exc}= 400 nm), c = 3×10^{-6} – 1.2×10^{-5} mol dm^{-3}; B: dependence of emission (λ_{em}= 530 nm) on *c*(**1**); C: emission spectra of **1** (λ_{em}= 530 nm); D: influence of temperature increase (T = 25°C – 95 °C) on fluorescence spectra of **1** ($c = 1.2\times10^{-5}$ mol dm^{-3}); E: ct-DNA addition. Performed in Na-cacodylate buffer, pH = 7.0, I = 0.05 mol dm^{-3}.

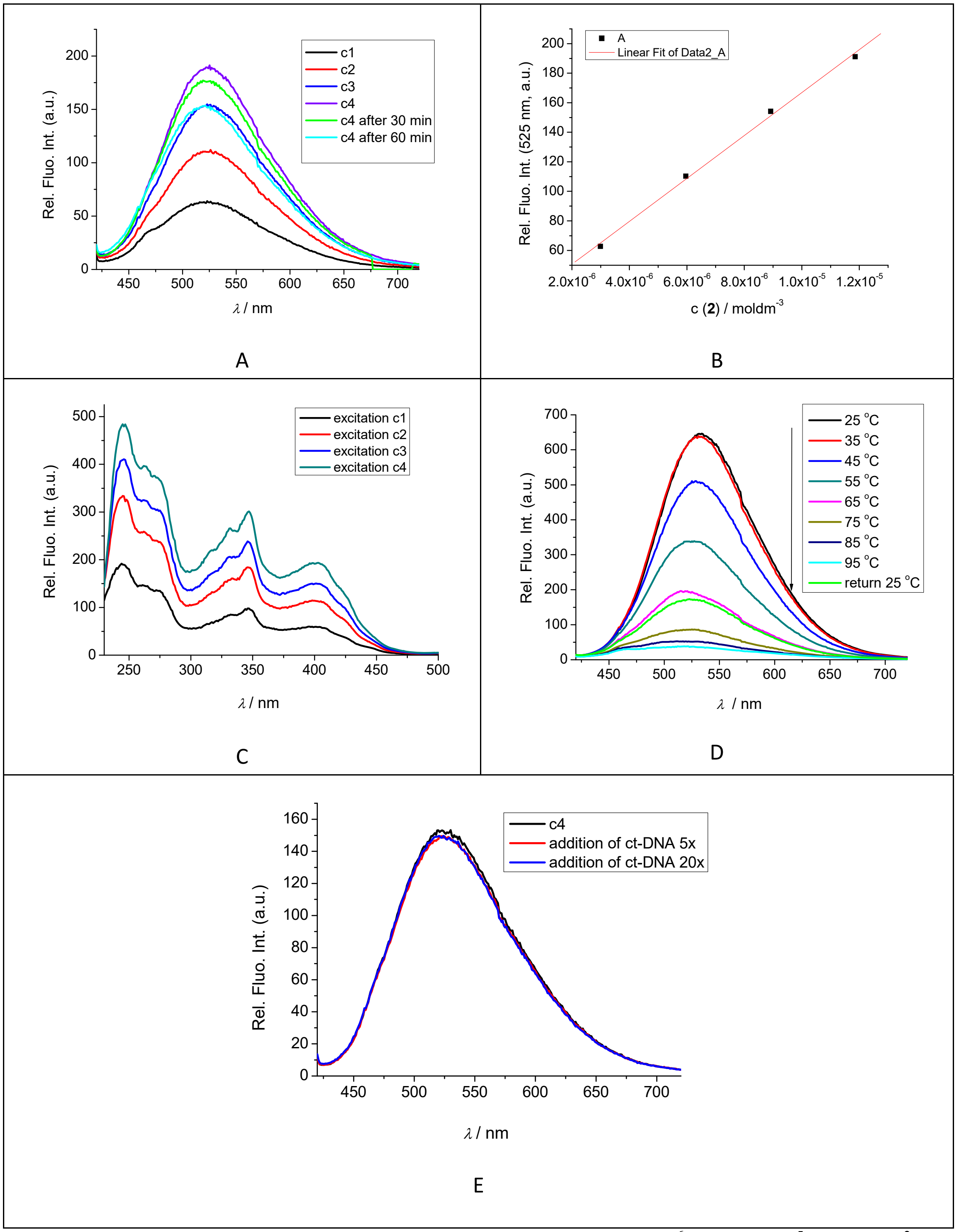

Figure S4. A: Emission spectra of **2** (λ_{exc}= 403 nm), c = 3×10^{-6} – 1.2×10^{-5} mol dm^{-3}; B: dependence of emission (λ_{em}= 525 nm) on *c*(**2**); C: emission spectra of **2** (λ_{em}= 525 nm); D: influence of temperature increase (T = 25°C – 95 °C) on fluorescence spectra of **2** ($c = 1.2\times10^{-5}$ mol dm^{-3}); E: ct-DNA addition. Performed in Na-cacodylate buffer, pH = 7.0, I = 0.05 mol dm^{-3}.

Fluorescence quantum yield was calculated according to:

$$\Phi_f = \Phi_{Ref} \left(\frac{n}{n_R}\right)^2 \frac{I}{I_R} \frac{1-10^{-A_R}}{1-10^{-A}} \qquad \text{(S1)}$$

Φ_f and Φ_{Ref} – fluorescence quantum yield of compound and the reference;
n and n_R – refractive index of the solvent in which compound or the reference was dissolved;
A and A_R – absorbance of the compound and the reference at the excitation wavelength;
I and I_R – area under emission curve of the compound and the reference

Fluorescence decays were fit to a sum of exponentials using the following expression:

$$F\,(t) = \alpha_1 \exp\left(-\frac{t}{\tau_1}\right) + \alpha_2 \exp\left(-\frac{t}{\tau_2}\right) + \alpha_3 \exp\left(-\frac{t}{\tau_3}\right) + \ldots \qquad \text{(S2)}$$

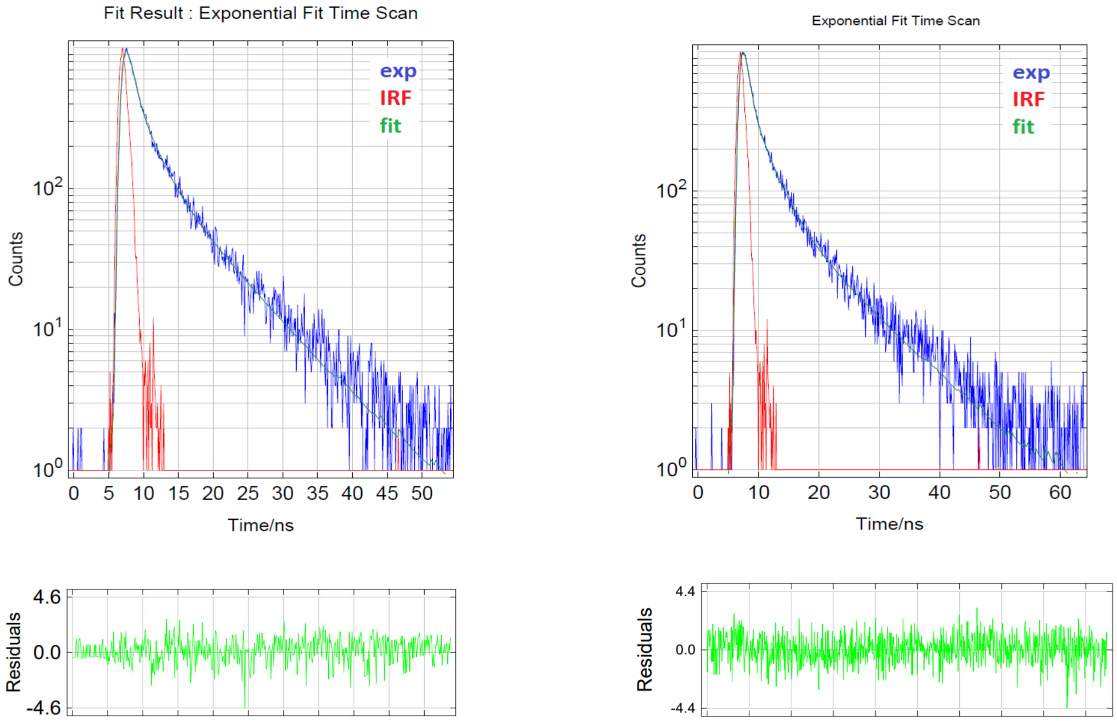

Fig S5. Decay of fluorescence for **1** (LEFT) and **2** (RIGHT) in aqueous medium at 530 nm upon excitation at 340 nm

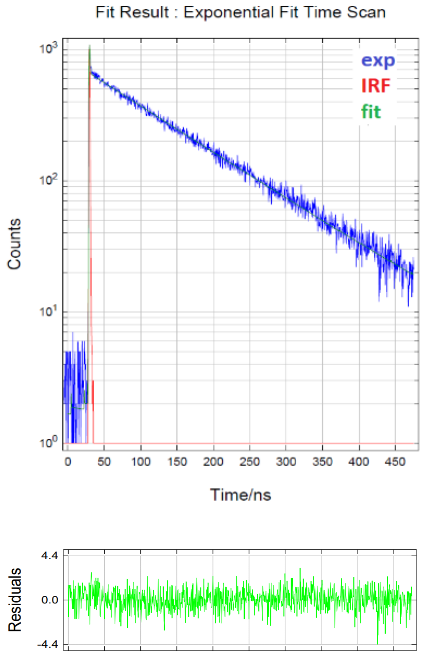

Fig S6. Decay of fluorescence for **2** in aqueous medium at 378 nm upon excitation at 340 nm

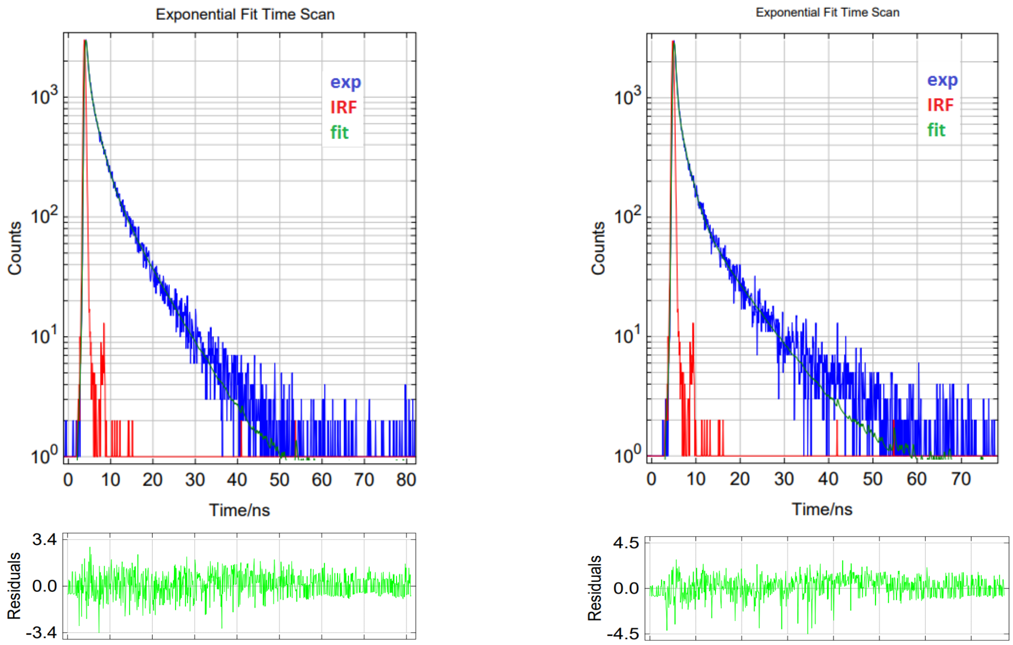

Fig S7. Decay of fluorescence for **1** (LEFT) and 2 (RIGHT) in aqueous medium at 530 nm upon excitation at 400 nm

Table S1. Fluorescence decay times and pre-exponential factors measured by TC-SPC for **1** and **2** in aqueous medium. The excitation wavelength was 340 or 400 nm, and the decays were collected at 378 and 530 nm.

Comp.	λ_{exc}	λ_{em}	τ / ns	Pre-exponential factors	$\chi 2$	τav / ns
**1**	340	530	0.50 ± 0.07<br>2.30 ± 0.22<br>7.80 ± 0.26	0.22<br>0.37<br>0.41	1.073	4.16
	400	530	0.33 ±0.02<br>2.01 ± 0.07<br>7.05 ± 0.14	0.21<br>0.45<br>0.34	0.805	3.39
**2**	340	378	0.18 ± 0.03<br>121 ± 0.43	0.01<br>0.99	1.069	119.8
		530	0.50 ± 0.05<br>2.84 ± 0.18<br>10.15 ± 0.42	0.29<br>0.40<br>0.31	0.896	4.43
	400	530	0.26 ± 0.01<br>1.83 ± 0.06<br>8.40 ± 0.20	0.33<br>0.41<br>0.26	1.095	3.02
**1-pyrene butyric acid**	340	419	7.23 ± 4.33<br>123.0 ± 0.32	0.0028<br>0.9972	1.041	122.68

At 340 nm the fluorescence is mostly detected from the pyrene. The decay of fluorescence for 1-pyrene butyric acid was fit to a sum of two exponents with the lifetime of τ^0 122.68 ns. Decay of fluorescence for **1** and **2** at 530 nm was fit to a sum of three exponents, while for **2** at 378 nm was fit to sum of two exponents. For the sum of exponents, the average lifetime was calculated according to:

$$\tau_{av} = \Sigma\alpha_i \tau_i \qquad \text{(S3)}$$

From the shortening of decay time at λ_{exc} = 340 nm for **2** at 530 nm, compared to the lifetime of 1-pyrene butyric acid, the efficiency of FRET (ΦFRET) can be calculated according to:

$$\Phi_{FRET} = 1 - \frac{\tau_{av}}{\tau^0} \qquad \text{(S4)}$$

Thermal denaturation experiments:

Thermal melting experiments were performed using a Varian Cary 100 Bio spectrometer in quartz cuvettes (1 cm). The measurements were done in aqueous buffer solution at pH 7.0 (sodium cacodylate buffer I = 0.05 mol dm^{-3}). Thermal melting curves for ds-DNA and complexes with **1** and **2** were determined by following the absorption change at 260 nm a function of temperature.[1] *Tm* values are the midpoints of the transition curves determined from the maximum of the first derivative and checked graphically by the tangent method. The Δ*Tm* values were calculated by subtracting *Tm* of the free nucleic acid from *Tm* of the complex. Every Δ*Tm* value reported here was the average of at least two measurements. The error in Δ*Tm* is ± 0.5 °C.

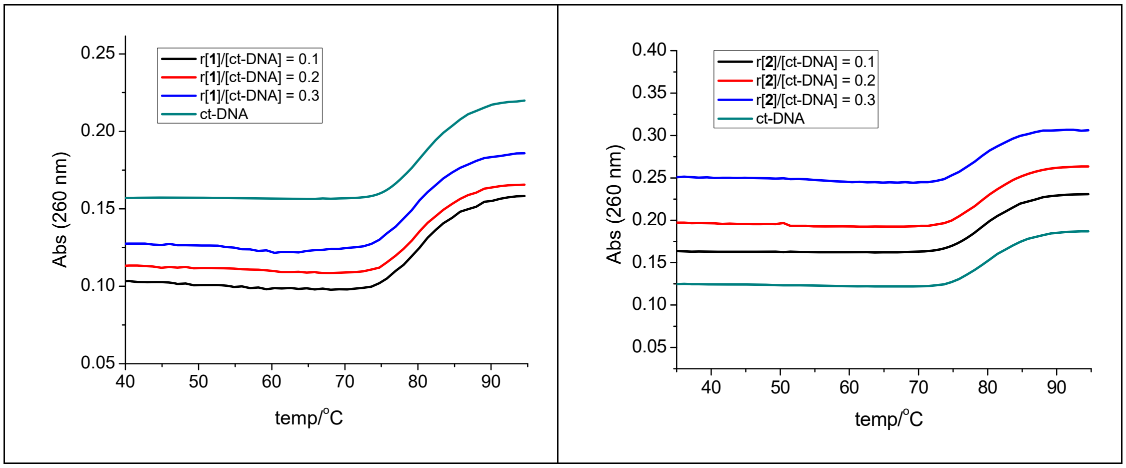

Figure S8. Thermal denaturation curves of **ct-DNA** upon addition of **1** (LEFT) and **2** (RIGHT). c(ct-DNA) = 2.5×10^{-5} mol dm^{-3}, $r_{[\mathbf{1,2}]/[\text{ct-DNA}]}$ = 0.1, 0.2, 0.3 at pH 7.0 (sodium cacodylate buffer, I = 0.05 mol dm^{-3}). Error in Tm values: 0.5 ºC.

Table S2. The [a]Δ*Tm* values (°C) of ct-DNA upon addition of ratio [b]***r*** of **1** and **2** at pH 7.0 (sodium cacodylate buffer, I = 0.05 mol dm^{-3})

[a]Δ*Tm*/°C	[b]r = 0.1	[b]r = 0.2	[b]r = 0.3
**compound**			
**1**	0	0	0
**2**	-1	-1	-1

[a] Error in ΔTm : ±0.5°C;

[b] r = [compound] / [polynucleotide];

Study of interactions with proteins

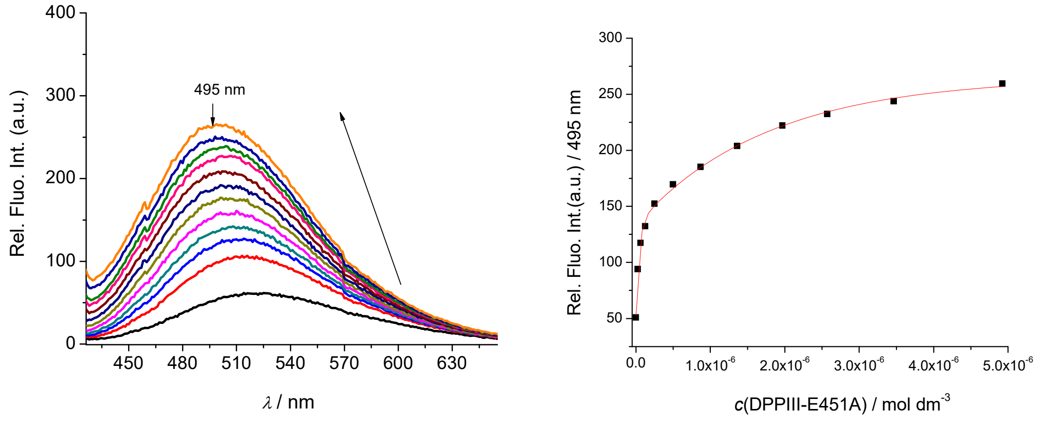

Figure S9. Fluorimetric titration of **2** ($c = 5 \times 10^{-7}$ mol dm^{-3}; λ_{exc} = 403 nm) with **DPP III-E451A**, in TRIS-HCl buffer at pH 7.4 , I = 0.02 mol dm^{-3}.

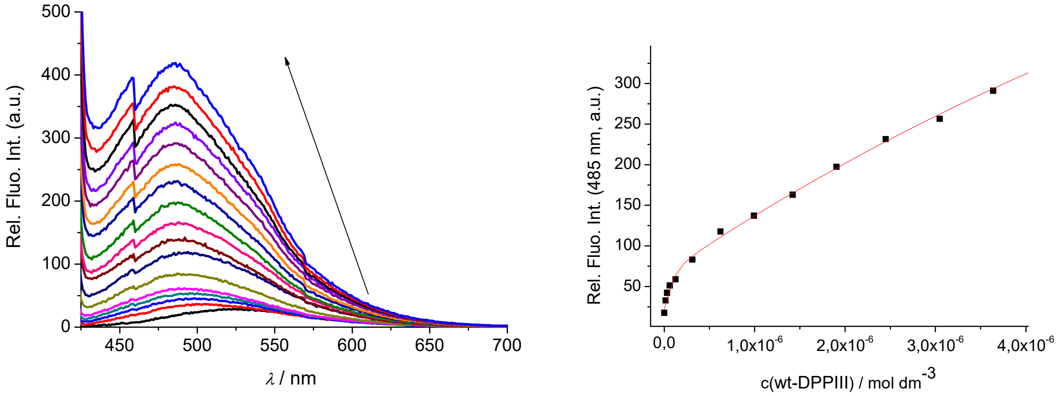

Figure S10. Fluorimetric titration of **1** (c = 5×10^{-7} mol dm^{-3}; λ_{exc} = 400 nm) with **wt-DPP III**, in TRIS-HCl buffer at pH 7.4, I = 0.02 mol dm^{-3}.

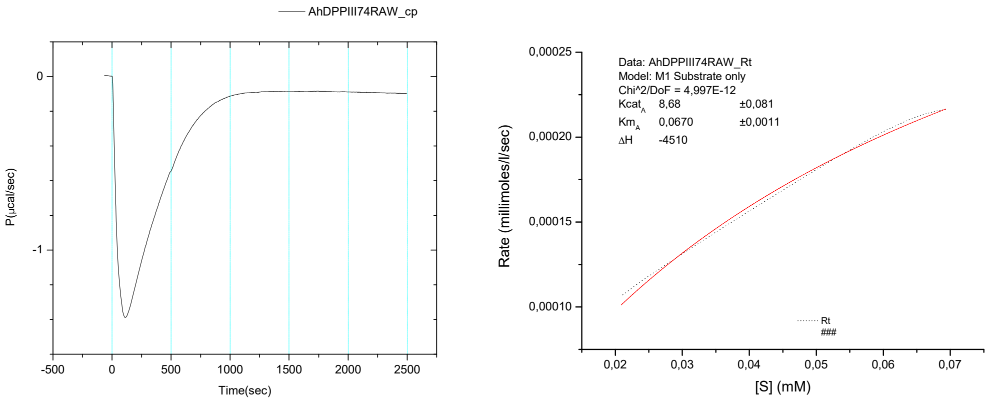

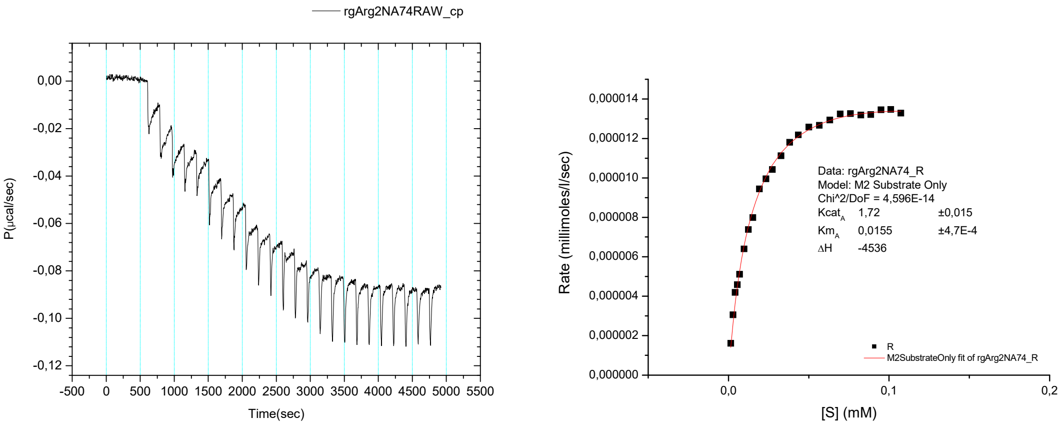

Figure S11. UP: Single injection ITC experiment, LEFT: Raw data of the enzyme reaction of wt-DPP III (c = 5x10^{-8} mol dm^{-3}) with the substrate Arg$_2$-2NA (c = 5x10^{-3} mol dm^{-3}), RIGHT: Fitted data of the enzyme reaction of wt-DPP III with the substrate Arg$_2$-2NA; DOWN: Multiple injection ITC experiment, LEFT: Raw data of the enzyme reaction of wt-DPP III (c = 1x10^{-8} mol dm^{-3}) with the substrate Arg$_2$-2NA (c = 1x10^{-3} mol dm^{-3}), RIGHT: Fitted data of the enzyme reaction of wt-DPP III with the substrate Arg$_2$-2NA.

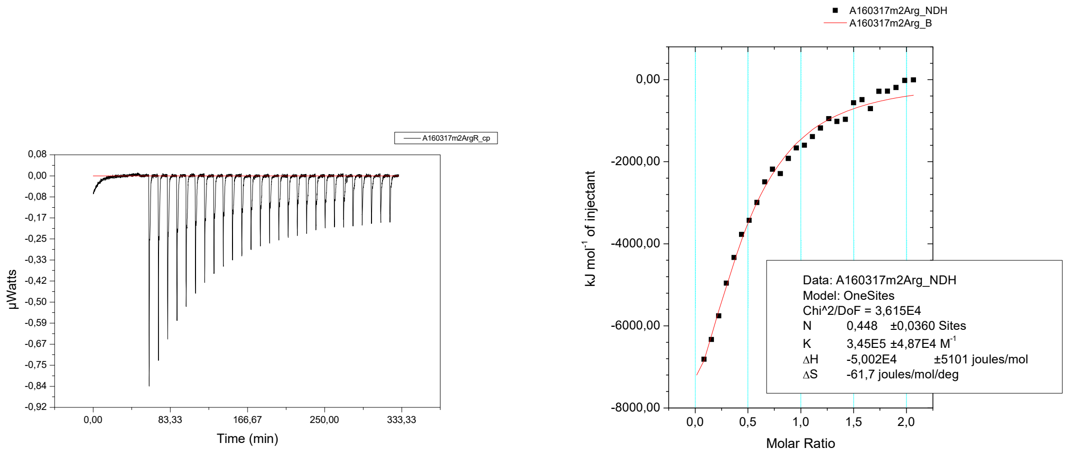

Figure S12. Multiple injection ITC experiment: Raw data (LEFT) and final figure for the ITC titration of DPP III-E451A with the substrate Arg$_2$-2NA (RIGHT)

DPP III kinetic assay

Table S3. Kinetic parameters for DPP III with and without **1**, calculated using nonlinear regression in GraphPad Prism5.

c (**1**), μM	K_m, μM	k_{cat}, s^{-1}
0	4.5 ± 0.5	7.5 ± 0.2
1	4.4 ± 0.8	7.8 ± 0.5
3	4.1 ± 0.5	8.5 ± 0.3

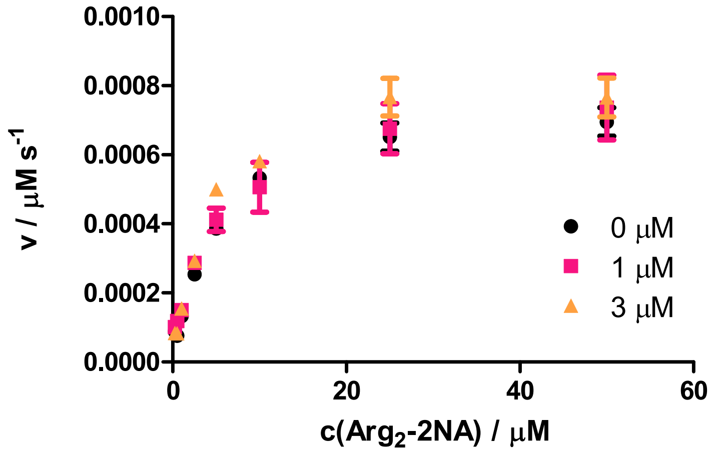

Figure S13. Michaelis-Menten plot of DPP III kinetics in the presence of 0 (black), 1 (pink), and 3 μM (orange) compound **1**.

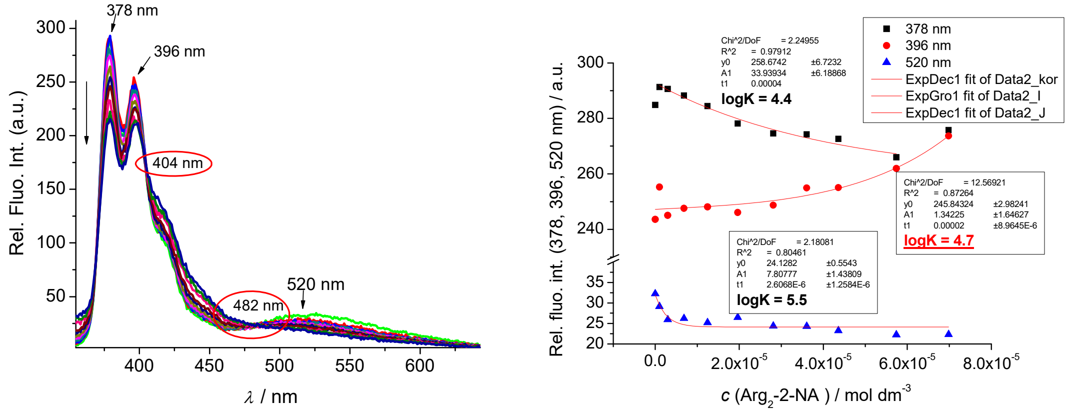

Figure S14. Fluorimetric titration of **2** (λ_{exc}= 342 nm, c = 1×10^{-6} mol dm^{-3}) with Arg$_2$-2NA (concentration range c = 1×10^{-6}– 7×10^{-5} mol dm^{-3}) in Tris-HCl buffer (pH 7.4, I = 0.02 mol dm^{-3}). Note isosbestic points at 404 nm and 482 nm, only one type of complex is formed.

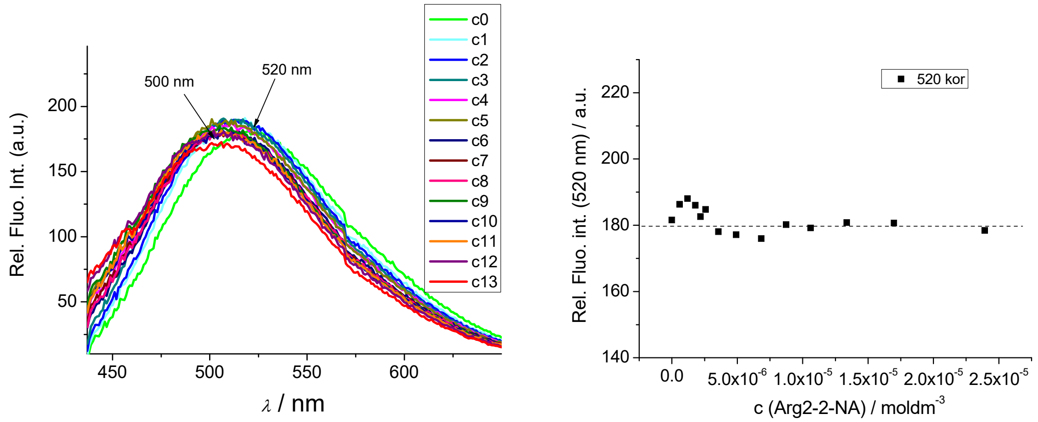

Figure S15. Fluorimetric titration of **2** (λ_{exc} = 403 nm, c = 1×10^{-6} mol dm^{-3}) with Arg$_2$-2NA (concentration range c = 6×10^{-7}– 2.4×10^{-5} mol dm^{-3}) in Tris-HCl buffer (pH 7.4, I = 0.02 mol dm^{-3}).

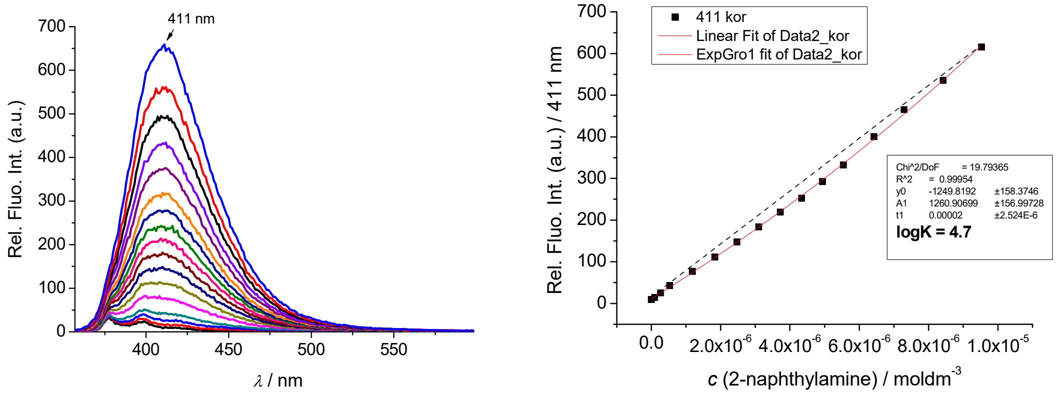

Figure S16. Fluorimetric titration of **2** (λ_{exc} = 342 nm, c = 1×10^{-6} mol dm^{-3}) with 2-naphthylamine (concentration range c = 1×10^{-7}– 1×10^{-5} mol dm^{-3}) in Tris-HCl buffer (pH 7.4, I = 0.02 mol dm^{-3}).

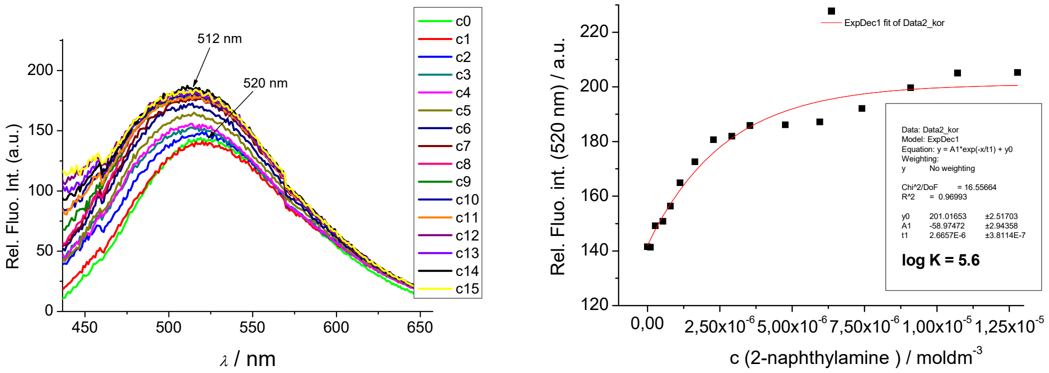

Figure S17. Fluorimetric titration of **2** (λ_{exc} = 403 nm, c = 1×10^{-6} mol dm^{-3}) with 2-naphthylamine (concentration range c = 1×10^{-7}– 1.2×10^{-5} mol dm^{-3}) in Tris-HCl buffer (pH 7.4, I = 0.02 mol dm^{-3}).

Molecular simulations

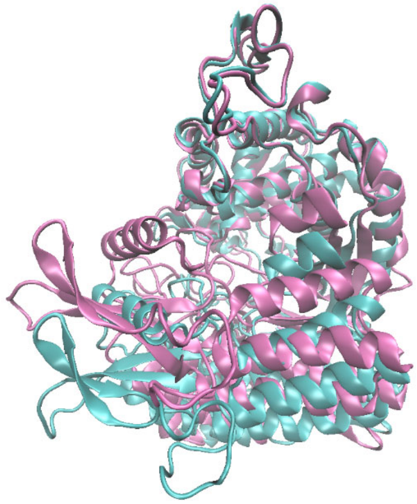

F

Figure S18. Overlap of two hDPP III forms used in the MD simulations: closed – mauve and semi-closed (partially open) – cyan.

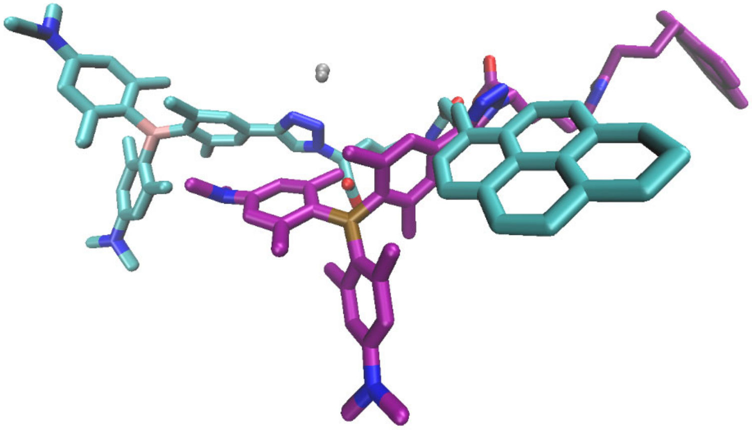

Figure S19. Two different initial binding modes of **2** in the overlapped enzyme binding sites considered in MD simulations. The structure with C atoms in cyan is named mod-1 and the

structure with C atoms in magenta mod-2. Hydrogen atoms were omitted for clarity. The Zn^{2+} ion in the enzyme active site is shown as a gray sphere.

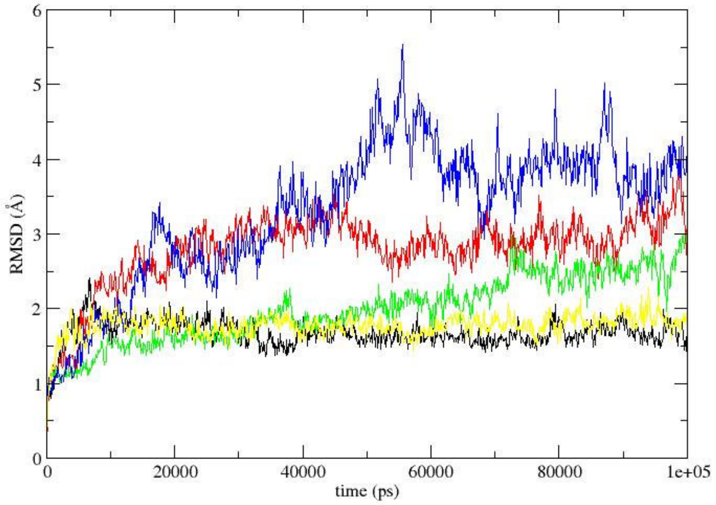

Figure S20. RMSD values calculated for the enzyme backbone atoms during RUNc-mod1 (r1 and r2), RUNc-mod2, RUNso-mod1 and RUNso-mod2 wherein values are coloured black, red, green, blue and yellow, respectively.

NOTE: RUNc-mod1 (r1 and r2), and RUNc-mod2 denote simulations of the hDPP III – **2** complex with hDPP III in its closed form with two different orientations of ligand, **2** (mod1 and mod2); RUNso-mod1 and RUNso-mod2 denote simulation of the hDPP III – **2** complex with hDPP III in its semi-closed form with the ligand bound in mod1 and mod2.

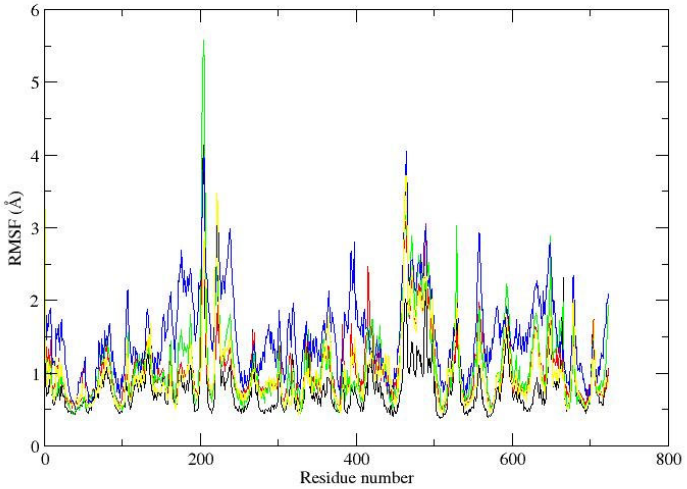

Figure S21. RMSF values calculated for the enzyme back bone atoms during RUNc-mod1 (r1 and r2), RUNc-mod2, RUNso-mod1 and RUNso-mod2 wherein values are coloured black, red, green, blue and yellow, respectively.

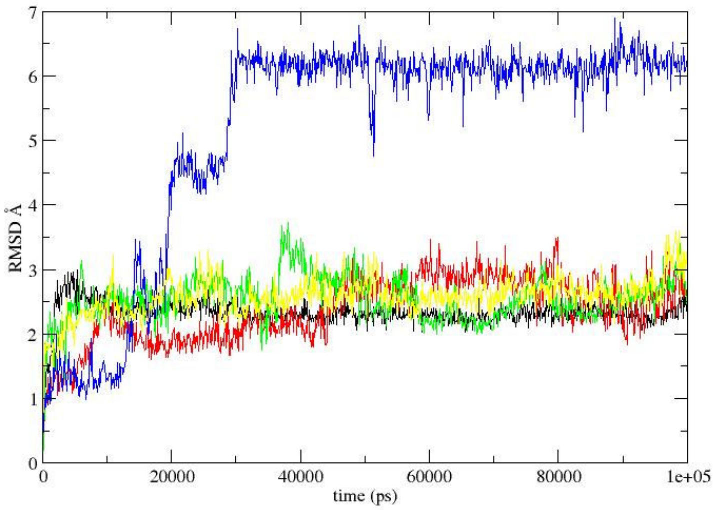

Figure S22. RMSD values calculated for the ligand atoms during RUNc-mod1 (r1 and r2), RUNc-mod2, RUNso-mod1 and RUNso-mod2 wherein values are coloured black, red, green, blue and yellow, respectively.

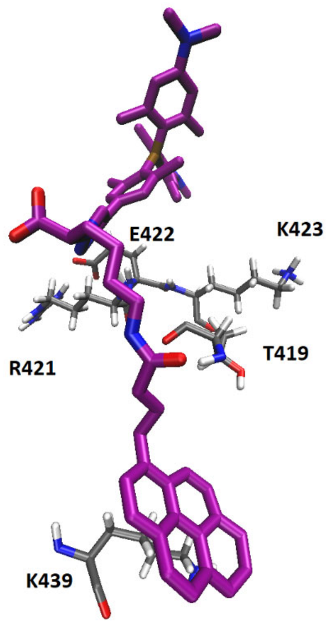

Figure S23. Stabilization of **2** (magenta) docked at the protein surface, with the hDPP III amino acid residues after 210 ns of MD simulations. Amino acid residues are coloured gray.

Table S4. MMGBSA energies calculated in 2 ns long intervals every 10 ns. RUNc-mod1 (r1 and r2) i RUNc-mod2 are simulations performed for the DPP III – **2** complexes with DPP III in the closed form, while RUNso-mod1 and RUNso-mod2 are simulation performed for the DPP III – **2** complexes with DPP III in semi-open form. The initial substrate orientation in RUNc-mod1 (r1 and r2) is the same as in RUNso-mod1, and in RUNc-mod2 as in RUNso-mod2.

E_{MMGBSA} (SD) kcal/mol				
RUNc-mod1-r1	RUNc-mod1-r2	RUNc-mod2	RUNso-mod1	RUNso-mod2
-101.0 (4.4)	-106.7 (5.5)	-67.9 (4.9)	-65.3 (6.4)	-47.6 (2.3)
-106.4 (7.8)	-111.8 (4.9)	-100.0 (8.7)	-62.9 (3.5)	-13.2 (5.6)
-106.7 (3.6)	-110.9 (5.3)	-77.0 (6.3)	-62.6 (5.3)	-34.6 (3.9)
-104.1 (5.8)	-107.4 (5.7)	-72.8 (7.1)	-67.9 (4.9)	-23.7 (2.4)
-95.3 (4.8)	-113.5 (5.9)	-58.1 (6.2)	-61.5 (5.0)	-22.9 (4.6)
-95.1 (5.3)	-113.7 (4.0)	-68.7 (6.4)	-61.1 (5.4)	-29.2 (3.9)
-89.3 (4.4)	-111.4 (4.1)	-66.2 (6.2)	-68.0 (5.2)	-31.6 (4.2)
-101.5 (7.6)	-113.9 (6.1)	-66.3 (6.1)	-67.6 (4.9)	-33.0 (3.8)
-92.5 (5.2)	-113.5 (4.0)	-64.6 (7.1)	-63.8 (6.2)	-23.8 (3.3)
-83.8 (5.7)	-112.9 (4.8)	-46.7 (6.3)	-68.9 (4.0)	-24.4 (3.4)

Table S5. Hydrogen bond population (%) during 100 ns of MD simulations of DPP III – **2** complexes.*

Residue	RUNc-mod1-r1	RUNc-mod1-r2	RUNc-mod2	RUNso-mod1	RUNso-mod2
Ser317					43
Tyr318	10	100	6		
Arg319			13		
Ala388		18			
Asn391					60
Asp396			54	9	
Asp397			11		
Arg399	100	100	93		
His455			15		
Trp495					9
Asp496			7		
Ser504			19	26	10
Glu508	9				
His568		24			29
Arg572		50			
Lys629			15		

*Default bond and angle cut-off values of 3.0 Å and 135°, respectively, were used to define hydrogen bonds. The hydrogen bond (HB) population is calculated as the ratio of the number of generated frames in which the bond is present and the total number of frames (HB_{pop} = N(frames with HB)/N(frames total)). In a case when a residue forms multiple hydrogen bonds, the hydrogen bond population is given as a sum of these values; if the sum exceeds 100, the value 100 is given in the Table. Interactions present in <5% of generated structures were omitted.

Table S6. MMGBSA energies calculated in 2 ns long intervals during the last 130 ns of the DPP III – **2** complexes with DPP III in the semi-open form and **2** bound at the protein surface.

Time (ns)	E_{MMGBSA} (SD) kcal/mol
84-86	-17.5 (2.1)
94-96	-17.9 (2.3)
98-100	-17.1 (3.8)
108-110	-16.2 (2.7)
118-120	-14.8 (4.9)
128-130	-23.7 (2.5)
138-140	-27.4 (4.1)
148-150	-33.6 (3.9)
158-160	-38.0 (4.0)
168-170	-37.2 (3.3)
178-180	-30.9 (3.6)
188-190	-33.8 (4.1)
198-200	-40.7 (3.5)
208-210	-35.7 (2.7)

Table S7. MMGBSA energies calculated in 2 ns long intervals during the 100 ns of the DPP III – RRNA – **2** complexes (I and II) with Arg-Arg-2NA bound into the enzyme active site and **2** bound at the protein surface.

Time (ns)	E_{MMGBSA} (SD) kcal/mol	
	DPP III – RRNA – 2 (I)	DPP III – RRNA – 2 (II)
8-10	-32.0 (3.6)	-30.2 (5.4)
18-20	-23.1 (2.6)	-25.3 (3.9)
28-30	-31.2 (5.2)	-26.0 (3.7)
38-40	-37.9 (3.5)	-32.3 (4.5)
48-50	-40.0 (4.1)	-15.8 (2.8)
58-60	-37.6 (3.3)	-18.6 (4.5)
68-70	-42.2 (3.4)	-7.6 (3.2)
78-80	-41.9 (3.5)	-22.2 (2.9)
88-90	-39.0 (4.8)	-19.1 (3.0)
98-100	-48.3 (3.3)	-27.9 (4.0)

NMR and HRMS Spectra

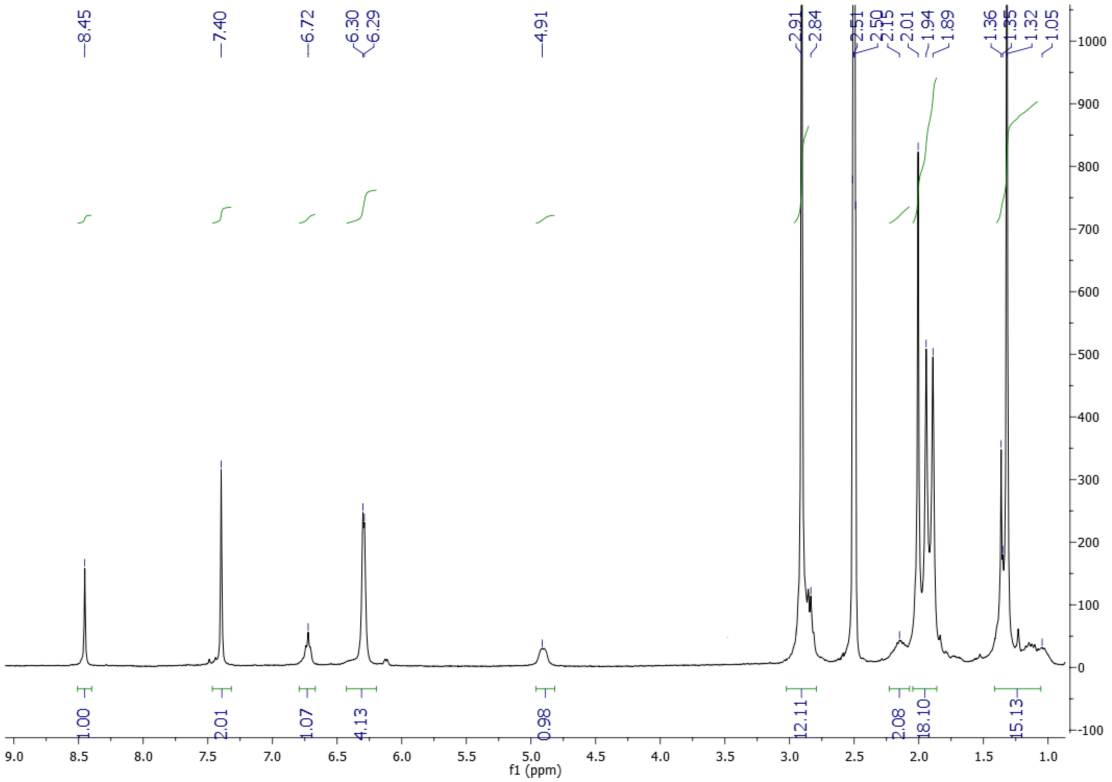

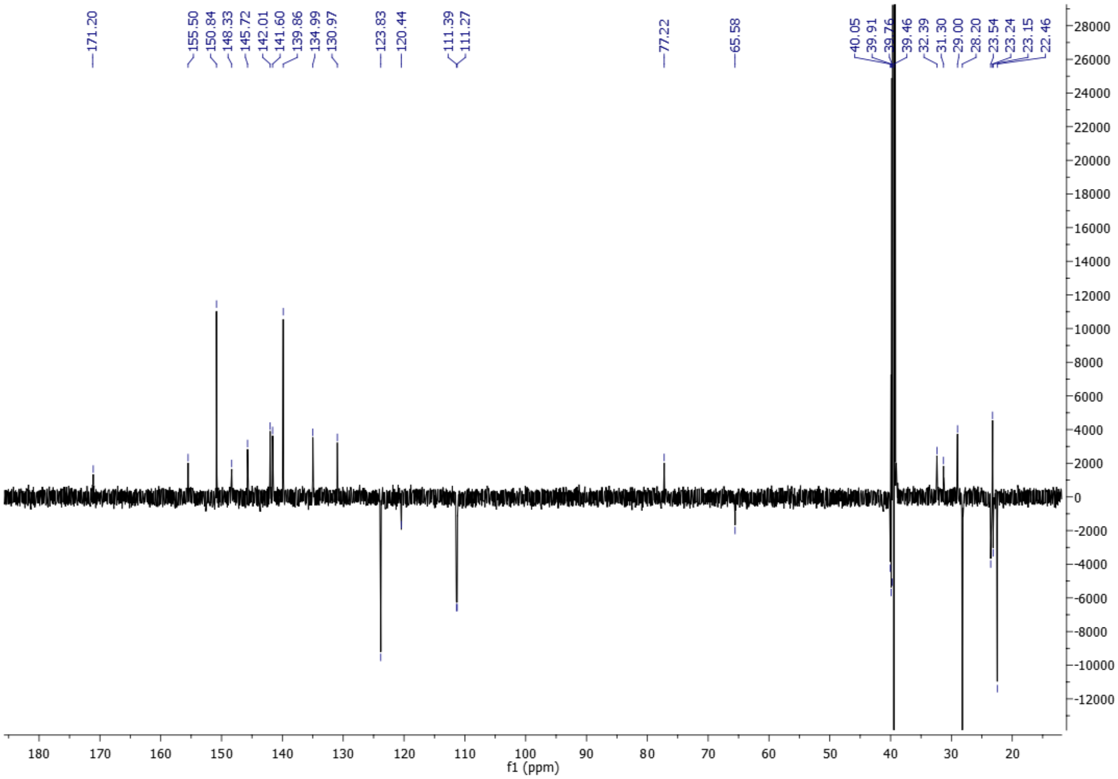

Figure S24. ^{1}H NMR (300 MHz, DMSO-d_6) and ^{13}C NMR (75 MHz, DMSO-d_6) spectra of compound C

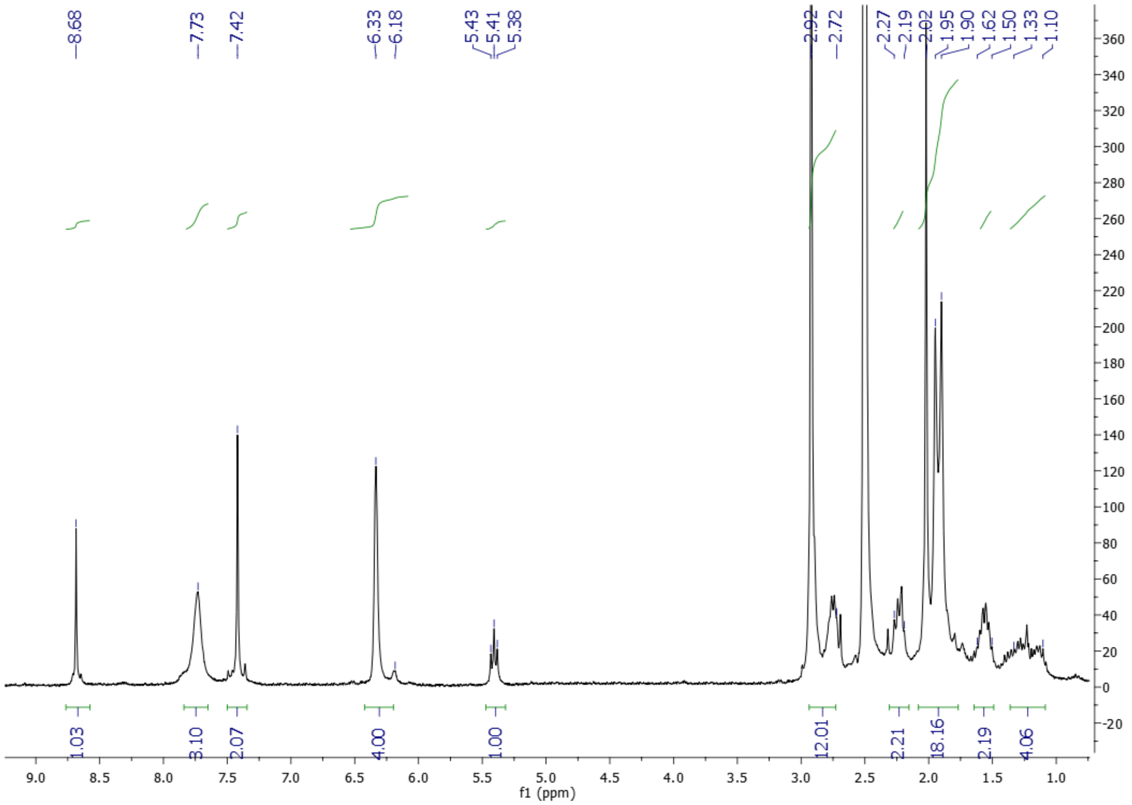

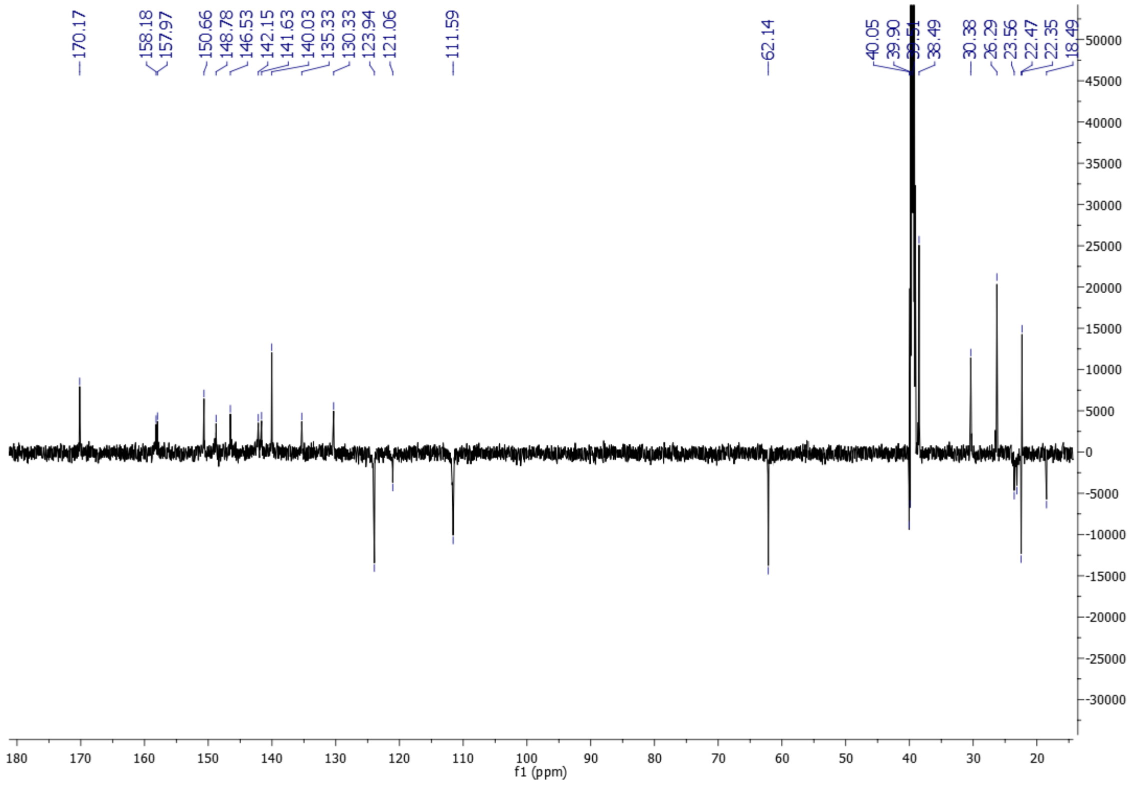

Figure S25. ^{1}H NMR (600 MHz, DMSO-d_6) and ^{13}C NMR (151 MHz, DMSO-d_6) spectra of compound **1**

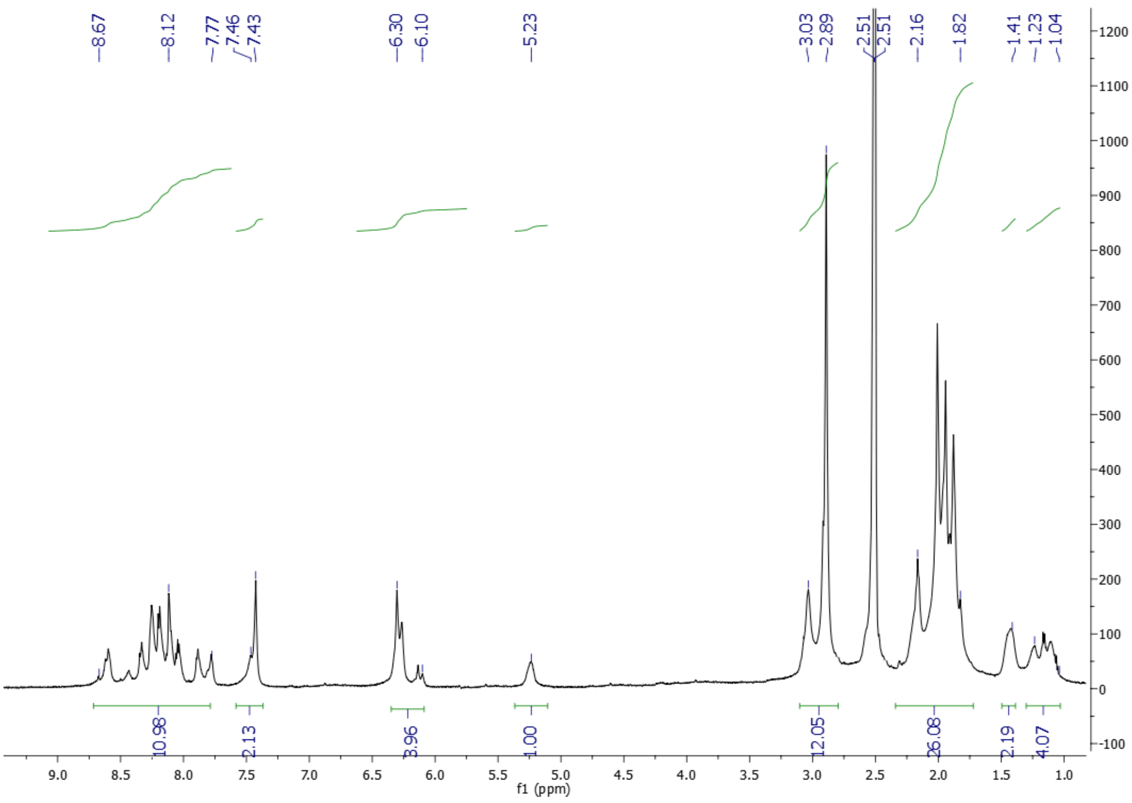

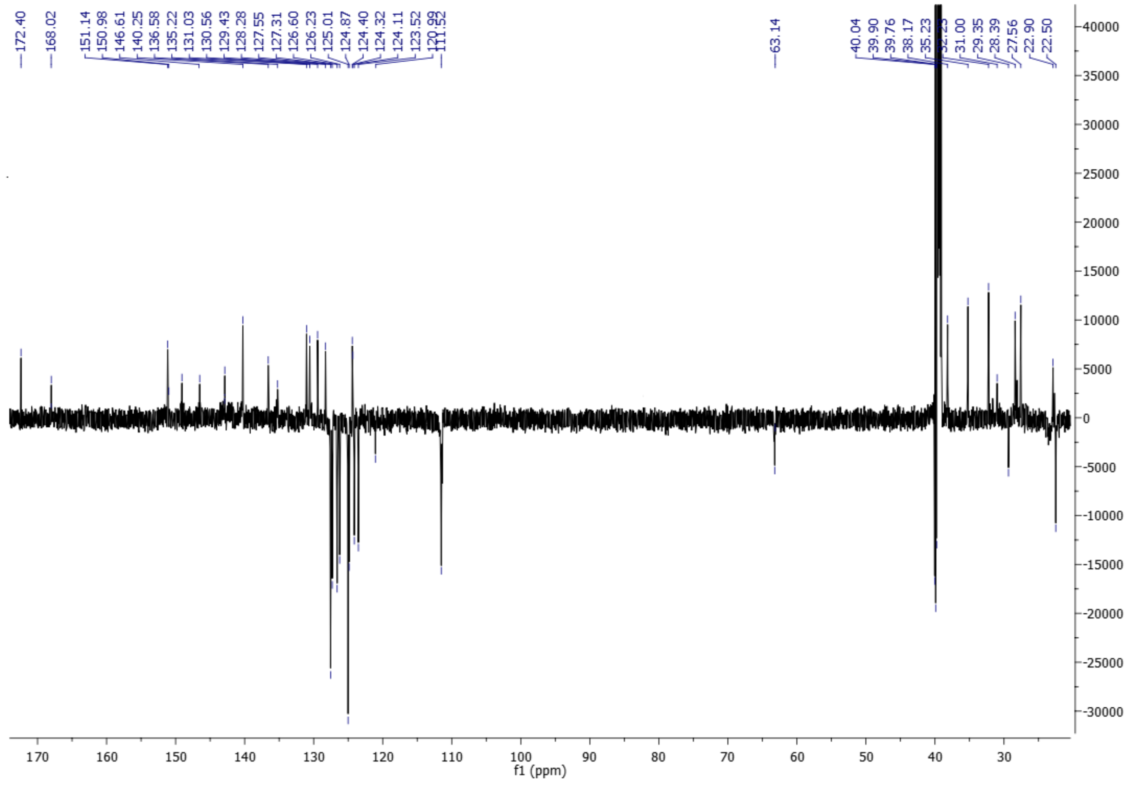

Figure S26. ^{1}H NMR (600 MHz, DMSO-d_6) and ^{13}C NMR (151 MHz, DMSO-d_6) spectra of compound **2**

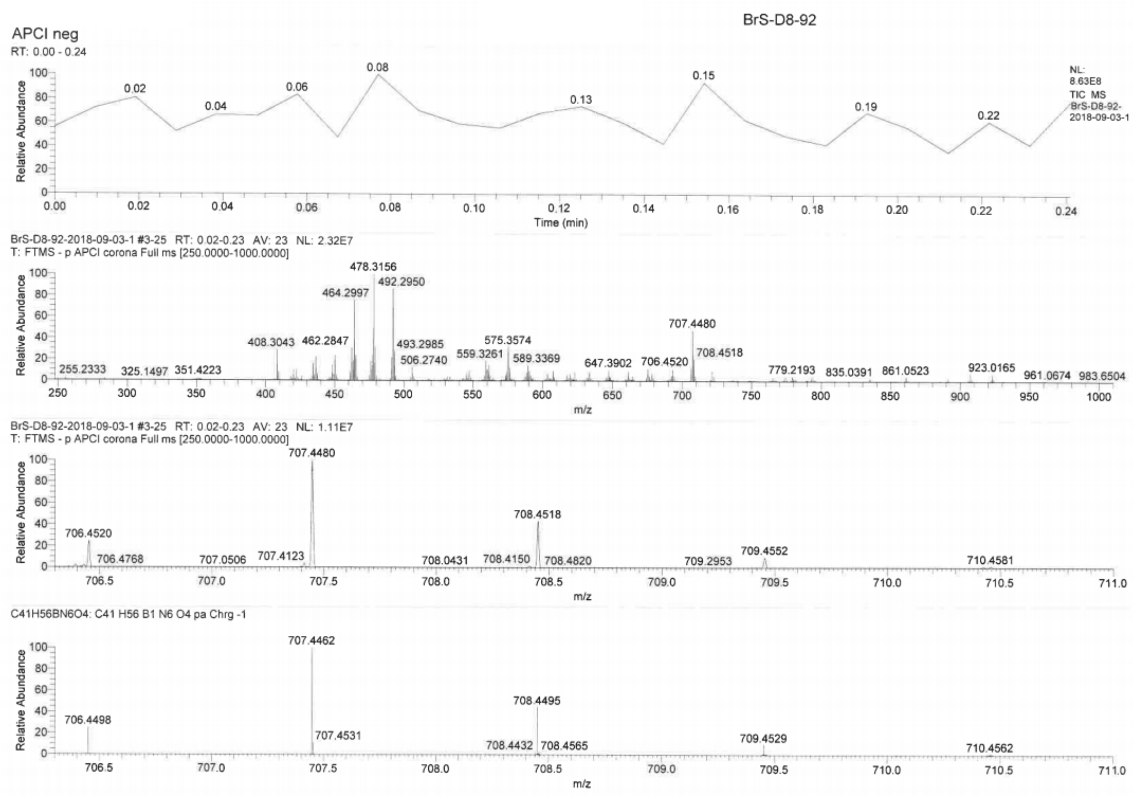

Figure S27. HRMS spectra of compound **C**

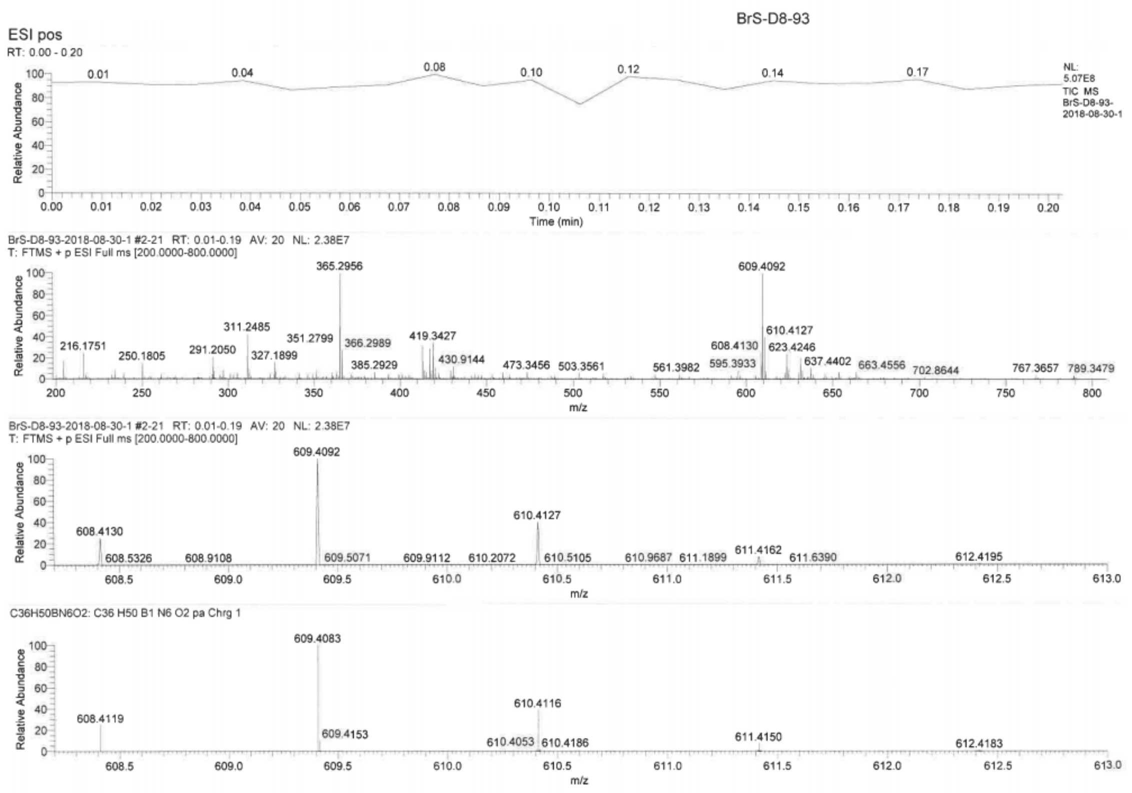

Figure S28. HRMS spectra of compound **1**

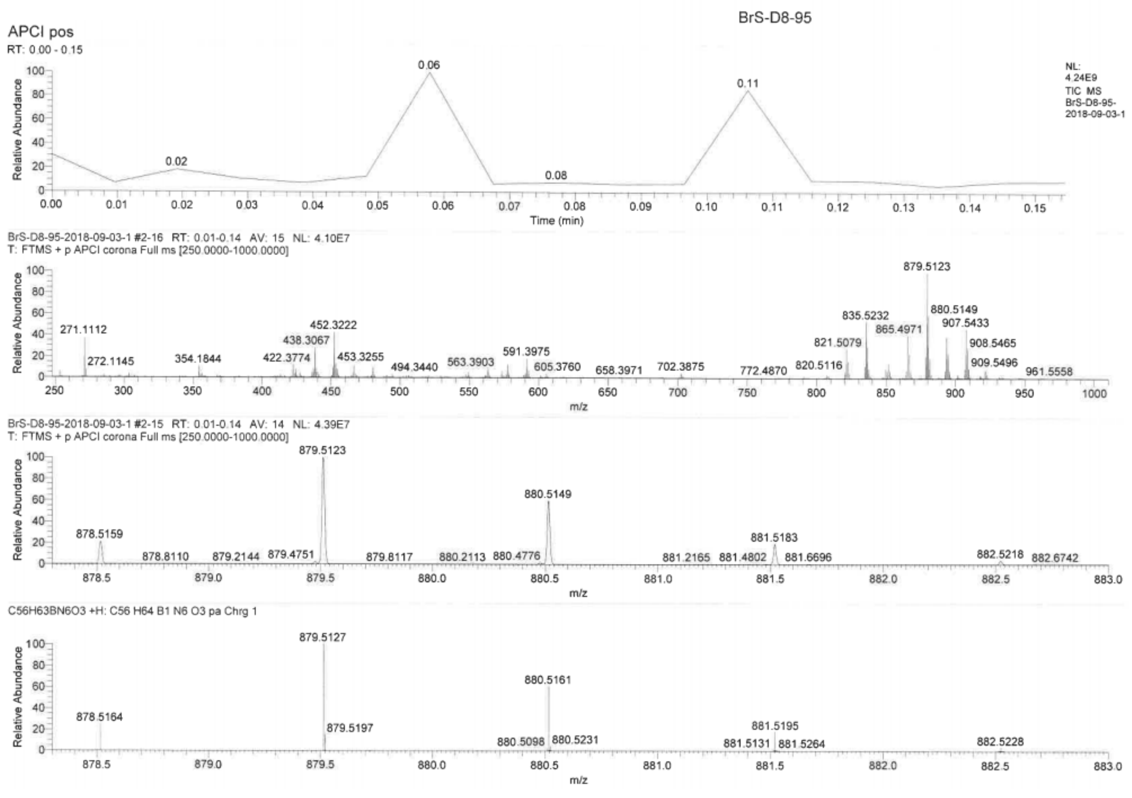

Figure S29. HRMS spectra of compound **2**

Reference:
1. J. L. Mergny, L. Lacroix, *Oligonucleotides* **2003**, *13*, 515-537.
